# Role of the dynamin-related protein 2 family and SH3P2 in clathrin-mediated endocytosis in *Arabidopsis thaliana*

**DOI:** 10.1242/jcs.261720

**Published:** 2024-05-02

**Authors:** Nataliia Gnyliukh, Alexander Johnson, Marie-Kristin Nagel, Aline Monzer, David Babić, Annamaria Hlavata, Saqer S. Alotaibi, Erika Isono, Martin Loose, Jiří Friml

**Affiliations:** ^1^ Institute of Science and Technology Austria (ISTA), 3400 Klosterneuburg, Austria; ^2^ Division of Anatomy, Centre for Anatomy & Cell Biology, Medical University of Vienna, 1090 Vienna, Austria; ^3^ Department of Biology, University of Konstanz, 78464 Konstanz, Germany; ^4^ Department of Biotechnology, College of Science, Taif University, Taif 21944, Saudi Arabia

**Keywords:** Clathrin-mediated endocytosis, Vesicle scission, Dynamin-related protein 2, SH3P2, Total internal reflection fluorescence microscopy, *Arabidopsis thaliana*

## Abstract

Clathrin-mediated endocytosis (CME) is vital for the regulation of plant growth and development through controlling plasma membrane protein composition and cargo uptake. CME relies on the precise recruitment of regulators for vesicle maturation and release. Homologues of components of mammalian vesicle scission are strong candidates to be part of the scission machinery in plants, but the precise roles of these proteins in this process are not fully understood. Here, we characterised the roles of the plant dynamin-related protein 2 (DRP2) family (hereafter DRP2s) and SH3-domain containing protein 2 (SH3P2), the plant homologue to recruiters of dynamins, such as endophilin and amphiphysin, in CME by combining high-resolution imaging of endocytic events *in vivo* and characterisation of the purified proteins *in vitro*. Although DRP2s and SH3P2 arrive similarly late during CME and physically interact, genetic analysis of the *sh3p123* triple mutant and complementation assays with non-SH3P2-interacting DRP2 variants suggest that SH3P2 does not directly recruit DRP2s to the site of endocytosis. These observations imply that, despite the presence of many well-conserved endocytic components, plants have acquired a distinct mechanism for CME.

## Teaser

**Highlighted Article:** In contrast to predictions based on mammalian systems, members of the plant dynamin-related protein 2 family are recruited to the site of clathrin-mediated endocytosis independently of BAR-SH3 proteins.

## INTRODUCTION

Clathrin-mediated endocytosis (CME) is a crucial cellular process that enables cells to respond to changes in the extracellular environment by internalising plasma membrane (PM), small molecules and transmembrane proteins. During CME, cargo is encapsulated within clathrin-coated vesicles (CCVs), which subsequently detach from the PM to undergo further trafficking, and cargo can undergo recycling or degradation (McMahon and Boucrot, 2011). CME plays a vital role in various cellular functions in plants, including cell wall synthesis, nutrient uptake, immune response and hormone signalling (Barberon et al., 2011; Bashline et al., 2013; Chen et al., 2011; Dhonukshe et al., 2007; Irani et al., 2012; Luschnig and Vert, 2014; Mbengue et al., 2016; Narasimhan et al., 2021; Paciorek et al., 2005; Pan et al., 2009; Postma et al., 2016; Sánchez-Rodríguez et al., 2018; Wang et al., 2017). CME regulates the internalisation of important PM proteins such as PIN-FORMED (PIN), Brassinosteroid Insensitive 1 (BRI1), borate receptor (BOR1), iron-regulated transporter 1 (IRT1) and cellulose synthase A (CESA) (Barberon et al., 2011; Bashline et al., 2013; Claus et al., 2018; Dhonukshe et al., 2007; Di Rubbo et al., 2013; Yoshinari et al., 2016; Zhang et al., 2019). Our understanding of CME mechanisms in plants originates mainly from mechanistic predictions made in mammalian and yeast cells, as plants (mainly the model *Arabidopsis thaliana*) possess homologous proteins to most key CME components including clathrin heavy chain (CHC) and clathrin light chain (CLC), adapter proteins (AP) and dynamin-related proteins (DRPs) (Dhonukshe et al., 2007; Gadeyne et al., 2014; Lu et al., 2016; McMahon and Boucrot, 2011). This led to the belief that plant CME works analogously to CME in other eukaryotic systems, although the physiological and biomechanical properties of plant cells are strikingly different from those of mammals and yeasts (Heidstra and Sabatini, 2014). The presence of a rigid cell wall and central vacuole creates high turgor pressure that influences mechanisms of various cell processes, including CME (Chen et al., 2011). Still, unlike in yeasts, in plants actin is not present at the site of endocytic vesicle formation (Narasimhan et al., 2020). Instead, a TPLATE complex, previously described to play the role of an adapter complex during CME (Gadeyne et al., 2014), has been suggested to be a plant-specific driver of membrane invagination (Johnson et al., 2021). These and other studies highlight significant differences between CME in plants and mammals and question its conserved mechanism (Backues et al., 2010; Fujimoto et al., 2010; Gadeyne et al., 2014). Therefore, to understand the mechanism of plant endocytosis, further investigation is needed.

During the final stage of CME, after the CCV has been fully formed, scission machinery mediates the release of the vesicle from the PM (McMahon and Boucrot, 2011). In mammalian systems, the essential step in CME is played by the large GTPase dynamin (Marks et al., 2001). It consists of a GTP hydrolysis (GTPase) domain, middle domain, GTPase effector domain (GED) and membrane binding pleckstrin homology (PH) domains followed by a protein–protein interaction proline-rich domain (PRD). This large GTPase assembles into oligomers around the highly curved membrane connecting the vesicle to the PM and releases the vesicle via GTP-hydrolysis-based conformational changes of the oligomer (Antonny et al., 2016). Thus, dynamin plays an important role in synaptic vesicle recycling and receptor-mediated endocytosis, sequestering ligands into invaginated coated pits (Perrais, 2022; Prichard et al., 2022). In yeasts, Vps1, Dnm1 and Mgm1 have been described as homologues of dynamin and they possess similar properties and function in CME (Lee et al., 2017; Rooij et al., 2010). They also have an N-terminal GTPase domain, middle domain and GED, but lack a PH domain and the canonical PRD. However, unlike mammalian dynamins, yeast homologues have been shown to play a role not only in vesicle fission, but also in the invagination and regulating the morphology of cortical actin patches (Rooij et al., 2010; Yu and Cai, 2004). In plants, many potential DRPs have been identified based on sequence homology and found to play a role in mitochondrial and chloroplast fission, during cytokinesis, cell plate formation and CME (Fujimoto et al., 2010; Lam et al., 2002). Specifically, members of the DRP1 and DRP2 subfamilies have been shown to colocalise with other endocytic markers on the PM. However, members of the DRP1 family have a domain organisation similar to that of their yeast homologues (lacking the PH domain and PRD), whereas the DRP2s have the same domain organisation as their mammalian counterparts (Hong et al., 2003). The DRP2 family members colocalise with CLC and proteins of the DRP1 subfamily on the PM (Fujimoto et al., 2010). The two members of the DRP2 subfamily, DRP2A and DRP2B, show a great sequence similarity and have previously been shown to be functionally redundant (Backues et al., 2010). Based on these observations, DRP2s have been thought to be involved in vesicle scission, but this assumption has not been definitely examined.

In mammals and yeast, the recruitment of dynamin to the high-curved membrane neck connecting the vesicle and the PM was found to be facilitated by bin-amphiphysin-Rvs (BAR) domain-containing proteins like endophilin (Endo2) and amphiphysin (Amph1) (Bhatia et al., 2009; Gallop et al., 2006; Pant et al., 2009; Renard et al., 2015). The BAR domain recognises high membrane curvature, and their Src homology-3 (SH3) domain interacts with other signalling and regulatory proteins (Peter et al., 2004; Xin et al., 2013). During membrane scission, the SH3 domain of Endo2 and Amph1 interacts with the PRD of dynamin, recruiting it to the site of the CME (Luo et al., 2016; Sundborger et al., 2014). Additionally, BAR domain-containing proteins can deform membranes, potentially aiding scission (Farsad et al., 2001; Peter et al., 2004). Disruption of the function of these proteins severely impairs synaptic vesicle endocytosis at central nerve terminals (Jockusch et al., 2005; Kontaxi and Cousin, 2023; Shupliakov et al., 1997). Thus, in mammalian cells the identity and regulation of several proteins required for vesicle formation during CME are well defined. In yeasts, although Vps1 lacks the canonical PRD, its interaction with the Amph1-homologue Rvs167 is important for vesicle scission, suggesting some degree of similarity between the mammalian and yeast systems (Smaczynska-de Rooij et al., 2012). Importantly, these studies emphasise the importance of BAR-domain-containing proteins for this process. However, whether this mechanism is conserved in plants is yet to be clarified.

Three members of the plant BAR-SH3-domain-containing protein family (SH3P1, SH3P2 and SH3P3) in *Arabidopsis* have been previously studied during cell plate assembly, endosomal sorting, intracellular trafficking and autophagosome biogenesis (Ahn et al., 2017; Baquero Forero and Cvrčková, 2019; Kolb et al., 2015; Nagel et al., 2017; Zhuang et al., 2013; Zhuang and Jiang, 2014). The importance of SH3P2 for vesicle trafficking [interaction with the endosomal sorting complexes required for transport (ESCRT)-I and -III system and ubiquitylated proteins in CCVs], autophagosome formation and cell plate formation has been demonstrated; however, little has been done to understand its role in the process of vesicle release from PM. Some studies have suggested involvement of SH3Ps in plant CME, where it is hypothesised that members of the SH3P family recruit the scission machinery to the clathrin-coated pit (CCP), in a similar way to their mammalian homologues such as Endo2 and Amph1 (Lam et al., 2001; Lebecq et al., 2022). For instance, SH3P proteins have been found to localise on clathrin-positive vesicles and colocalise with Auxilin-LIKE1, which likely participates in the uncoating of CCVs (Adamowski et al., 2024; Dahhan et al., 2022; Nagel et al., 2017). Notably, the SH3 domain of SH3P3 interacts with members of the DRP2 family (Lam et al., 2002). Additionally, the BAR domain of SH3P2 has been shown to bind and tubulate liposomes *in vitro* (Ahn et al., 2017)*.* Although these findings suggest involvement of SH3P proteins in plant CME, a comprehensive characterisation of their behaviour and properties *in vivo* and *in vitro* is currently missing. This lack of knowledge makes it difficult to propose a mechanism for plant CME and the roles of the proteins involved.

In this study, we aimed to obtain new insights into plant CME, focusing specifically on the roles of DRP2s and SH3P2. By using high-resolution imaging *in vivo,* we found that SH3P2, similar to DRP2A and DRP2B, arrives at the PM at the end of the endocytic event. In *in vitro* experiments, we found that purified SH3P2 is able to deform membranes, as previously described for mammalian homologues. Our data further reveal colocalisation *in planta* and direct interaction between purified SH3P2 and DRP2B *in vitro*. We found that PM internalisation is significantly impaired in the *sh3p123* triple mutant, whereas DRP2A, CLC2 and TPL endocytosis markers show normal dynamics. Thus, our findings shed light on the dynamics of two key players in the predicted plant endocytic scission machinery, suggesting that DRP2s have a distinct recruiting mechanism from their mammalian counterparts that is largely independent of SH3P2.

## RESULTS

### DRP2 arrives at the end of the endocytic event

Proteins involved in endocytosis show a stereotypical timing of recruitment depending on their function (McMahon and Boucrot, 2011). For example, regulators involved in vesicle scission, like dynamin, Endo2, and Amph1, are recruited to endocytic events right before the vesicle is pinched off and released from the PM (Rosendale et al., 2019; Taylor et al., 2011). Given the homology of DRP2B to dynamins, and that it previously found to colocalise with CLC at the PM (Fujimoto et al., 2010), we decided to obtain more information on their dynamics together with CME markers *in planta* at high spatiotemporal resolution to gain insights into role of DRP2s in plant endocytosis.

We used total internal reflection fluorescence microscopy (TIRF-M) of epidermal root cells, which allowed us to create high-resolution time-lapse images of protein dynamics exclusively on the PM (Johnson et al., 2020; Trache and Meininger, 2008). Before imaging, we confirmed that DRP2A tagged with C-terminal GFP was functional by checking that it could complement the ovule phenotype of *drp2a-1−*/*−;drp2b-2+*/*−* heterozygous double mutant (Fig. S1A,B) (Backues et al., 2010). Additionally, TIRF-M of DRP2A–GFP in *drp2a-1−*/*−;drp2b-2−*/*−* (Fig. S1C–F) showed an increase in dynamics and spot density compared to the control (DRP2A–GFP in *drp2a-1−*/*−*) (Fig. S1G–I), potentially indicating a compensatory mechanism for lack of DRP2B.

**Fig. 1.**
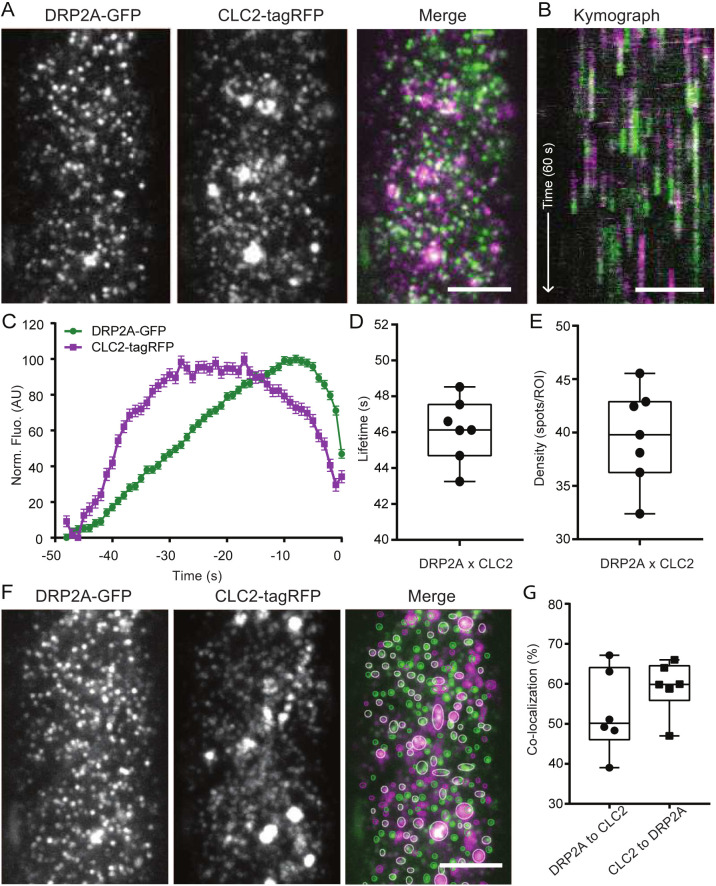
**Recruitment of DRP2A to the site of vesicle formation.** (A) TIRF-M images of a cell surface of root epidermal cell expressing *pDRP2A::DRP2A-GFP* and *pRPS5A::CLC2-tagRFP* (*drp2a-1−/−*). (B) Representative kymograph of DRP2A and CLC2 lifetimes on the PM. The arrow represents the time direction and the length of 60 s. (C–E) Data from seven independent experiments were combined to generate the (C) mean±s.e.m. recruitment profile of DRP2A to the site of endocytosis; (D) mean±s.e.m. lifetime of CME events, 46.12±0.6 s; and (E) mean density of CME events, 39.63 spots per ROI. *n*=7 cells from independent roots, 22,432 tracks. (F) Representative image of colocalisation analysis of DRP2A and CLC2 foci. The circled areas indicate detected spots (green and magenta circles for GFP and tagRFP, respectively). White circles represent detections in both channels. (G) Quantification of colocalised spots. 52.98% (mean) of DRP2A was colocalised to CLC2, and 59.25% of CLC2 was colocalised to DRP2A. *n*=7 cells from independent roots. For box plots in D, E and G, the box represents the 25–75th percentiles, and the median is indicated. The whiskers show the maximum to minimum range. AU, arbitrary units. Scale bars: 5 µm (A,B,F).

To analyse the recruitment of DRP2A exclusively within CME events, we simultaneously visualised DRP2A–GFP and CLC2 fluorescently tagged with tagRFP, a CME marker, and applied an automated analysis to obtain the precise arrival and departure times of the protein at the PM (Fig. 1A) (Johnson et al., 2020). Kymographs can visualise the persistence of the fluorescent foci by creating a projection of a random *x*-plane over the time span of the timelapse image (Fig. 1B). Analysis of fluorescent profiles of DRP2A colocalised with CLC2 showed that the maximum intensity of DRP2A recruitment occurred before the CLC2 signal disappeared, marking the departure of the vesicle from PM (Fig. 1C). The average lifetime of these events was 46.3±0.22 s (mean±s.e.m.) with a density of 39.64 spots per region of interest (ROI; each ROI is 100×100 pixels) (Fig. 1D,E), compared to a long population of endocytosis events represented by TPLATE (TPL) and CLC2 (∼43 s) (Narasimhan et al., 2020). Our data showed that 60% of CLC2 foci colocalised with DRP2A (Fig. 1F,G), suggesting that a significant amount of CME foci include DRP2A. We observed similar results for the second member of the DRP2 family, DRP2B–GFP with CLC2–tagRFP (Fig. S2).

The observed recruitment profile of DRP2A is typical for proteins involved in the late stages of CCV formation, such as during scission. Therefore, these observations support the hypothesis that DRP2 proteins are part of the CME scission machinery.

### SH3P2 arrives at the end of endocytosis, similar to DRP2

Recruitment of dynamin and Vps1 are tightly linked to the arrival of Amph1/Endo2 and Rvs167 to the site of CCV formation in mammals and yeast, respectively. Previously, the plant homologue of these proteins, SH3P2, has been shown to colocalise with CLC and co-fractionate with CCVs, providing initial indications for its function in CME (Adamowski et al., 2024; Dahhan et al., 2022; Nagel et al., 2017).

**Fig. 2.**
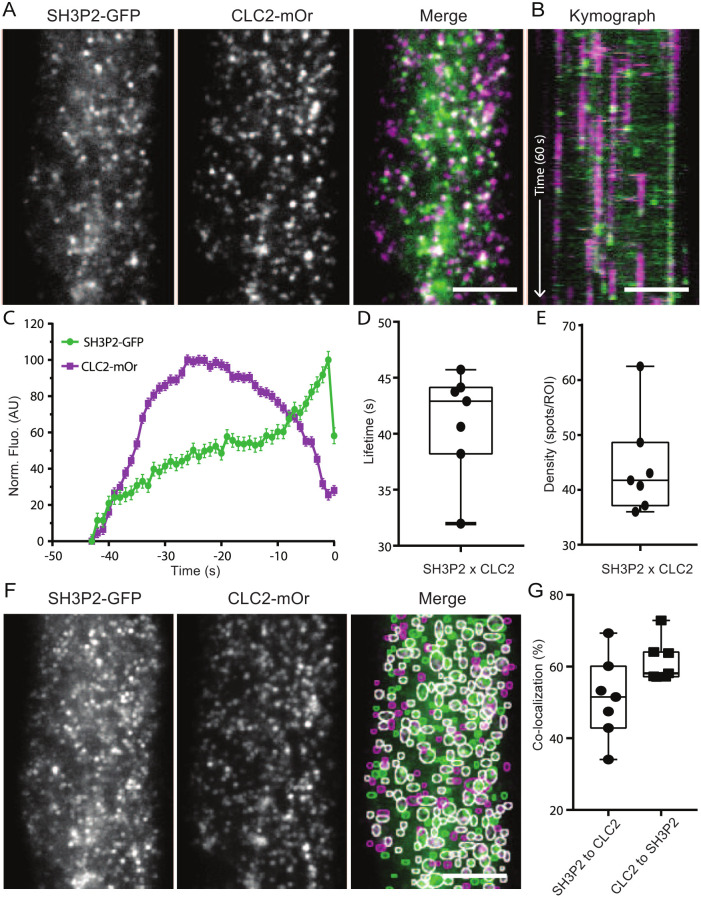
**SH3P2 arrives at the end of the endocytosis event.** (A) TIRF-M images of a cell surface of root epidermal cell expressing *pRPS5A::SH3P2-sGFP* and *pRPS5A::CLC2-mOrange*. (B) Representative kymograph of SH3P2 and CLC2 lifetimes on the PM. Arrow represents the time direction and timespan of 60 s. (C–E) Data from seven independent experiments were combined to generate the (C) mean±s.e.m. recruitment profile of SH3P2 to the site of endocytosis; (D) mean±s.e.m. lifetime of CME events, 41.05±0.5 s; and (E) mean density of CME events, 44.26 spots per ROI. *n*=7 cells from independent roots, 24,170 tracks. (F) Representative image of colocalisation analysis of SH3P2 and CLC2 foci. The circled areas indicate detected spots (green and magenta circles for GFP and tagRFP, respectively). White circles represent detections in both channels. (G) Quantification of colocalised spots. 51.25% (mean) of SH3P2 was colocalised to CLC2, and 61.49% of CLC2 was colocalised to SH3P2. *n*=7 cells from independent roots. For box plots in D, E and G, the box represents the 25–75th percentiles, and the median is indicated. The whiskers show the maximum to minimum range. AU, arbitrary units. Scale bars: 5 µm (A,B,F).

To investigate the function of SH3P2 in plant CME, we quantified the recruitment of SH3P2 tagged with superfolder GFP (sGFP) compared to CLC2–mOrange, used as a reference for CME events. We analysed individual endocytosis events positive for both CLC2 and SH3P2. The peak of the SH3P2–sGFP signal occurred just before the drop of the CLC2-mOrange signal, indicating of CCV scission (Fig. 2A,B). The average lifetime of SH3P2- and CLC2-positive events was 40.54±0.19 s with an average density of 44.26 spots per ROI (mean) (Fig. 2C–E), which is in line with data obtained for DRP2s and the lifetime for a bona fide population of endocytosis events reported in Narasimhan et al. (2020). Next, the colocalisation frequency of CLC2 and SH3P2 at a given time point was 60% of the CLC2, similar to what we had observed for DRP2A colocalisation with CLC2 (Fig. 2F,G). The profile of SH3P2 recruitment to the site of CCV formation suggests that SH3P2 functions at the final stage of CME in plants.

These *in vivo* observations reveal that both SH3P2 and DRP2A are specifically recruited at the end of the CME events on the PM, much like their homologues during CME in other systems.

### SH3P2 binds and bends membranes *in vitro*

Complementation of protein dynamics in live cells with *in vitro* experiments of individual proteins provides a more detailed and controlled understanding of their function. Therefore, to study the properties of DRP2s and SH3P2, we decided to test their binding, bending and fission abilities using purified proteins *in vitro*. Unfortunately, after extensive efforts, we were not able to purify GTPase-active full-length DRP2A or DRP2B. It is known that GTPase activity is crucial for its function *in vivo*, and therefore we continued the characterisation of SH3P2 protein alone.

In mammalian systems, the BAR-SH3-domain-containing proteins Endo2 and Amph1 bind to and remodel membranes, which is crucial for the successful vesicle formation (Blood and Voth, 2006; Habermann, 2004). Previous studies have demonstrated that the isolated BAR domain of *Arabidopsis* SH3P2 predominantly binds negatively charged membranes and induces vesicle deformation *in vitro* (Ahn et al., 2017).

To assess the membrane binding and remodelling capacity of SH3P2, we purified the bacterially expressed protein (Fig. S3A). Mass photometry measurements showed two peaks in the histogram corresponding to the mass of the SH3P2 monomer (∼39 kDa) and dimer (∼78 kDa) (Young et al., 2018). With an increase of protein concentration in solution from 75 nM to 100 nM, we observed an increase percentage of dimers (60% versus 85%), suggesting that dimerisation is concentration dependent (Fig. S3B). This confirms that purified SH3P2, similar to BAR-domain-containing proteins from mammalian system, can dimerise (Jhaveri et al., 2021; Youn et al., 2010).

**Fig. 3.**
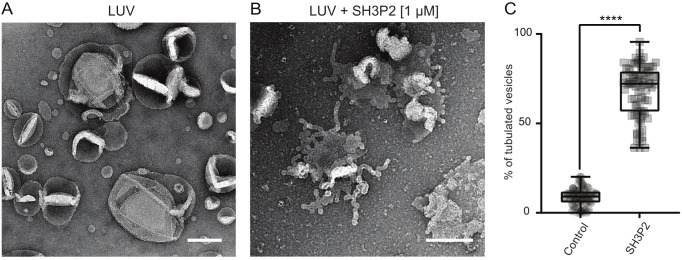
**Full-length SH3P2 protein bends membranes *in vitro*.** (A,B) Example TEM overviews of LUVs after 5 min incubation in control conditions (A) or with 1 µM SH3P2 (B). Scale bars: 200 nm. (C) Quantification of the percentage of LUVs that displayed tubulation. 8.83% of LUVs displayed tubulation in control conditions. 68.02% of LUVs incubated with SH3P2 displayed tubulation. Control, *n*=77 images and SH3P2, *n*=85 images pooled from three independent experiments. The box represents the 25–75th percentiles, and the median is indicated. The whiskers show the maximum to minimum range. ****P<0.001 (unpaired two-tailed *t*-test to compare to control).

In mammalian systems, the cooperative membrane binding of BAR-domain-containing proteins and dynamin is important for vesicle release from the PM (Meinecke et al., 2013). Therefore, we wanted to understand the ability of SH3P2 to bind membranes, and hence conducted a liposome sedimentation assay, where the protein of interest only sediments when bound to phospholipid vesicles. As it has been shown that sites where CME occurs at the PM are enriched with negatively charged lipids (Martin, 2001), we decided to study the lipid binding preferences of SH3P2. We generated large unilamellar vesicles (LUVs) of different lipid compositions, with and without negatively charged lipids, such as phosphatidic acid (PA) and phosphatidylinositol 4,5-bisphosphate [PI(4,5)P_2_]. We found that SH3P2 exhibited a preference for binding to LUVs containing PA and even more PI(4,5)P_2_ compared to LUVs with only 1,2-dioleoyl-sn-glycero-3-phosphocholine (DOPC) or DOPC mixed with 1,2-dioleoyl-sn-glycero-3-phospho-L-serine (DOPS) (Fig. S3C,D). Finally, to test the membrane deforming capability of SH3P2, we incubated the purified proteins with LUVs with DOPC, DOPS and PI(4,5)P_2_ and performed transmission electron microscopy (TEM) experiments. Compared to the control vesicles without protein, LUVs showed a significant percentage of membrane deformation in presence of SH3P2 (Fig. 3), demonstrating the ability of the protein to deform membranes.

In conclusion, reconstitution experiments *in vitro* show that SH3P2, similar to its mammalian homologues, dimerises and preferentially binds negatively charged lipids. Moreover, similarly to the BAR domain of SH3P2 alone, full-length SH3P2 promotes tubulation of LUVs *in vitro*.

### DRP2A and SH3P2 interact *in vitro* and colocalise *in vivo*

**Fig. 4.**
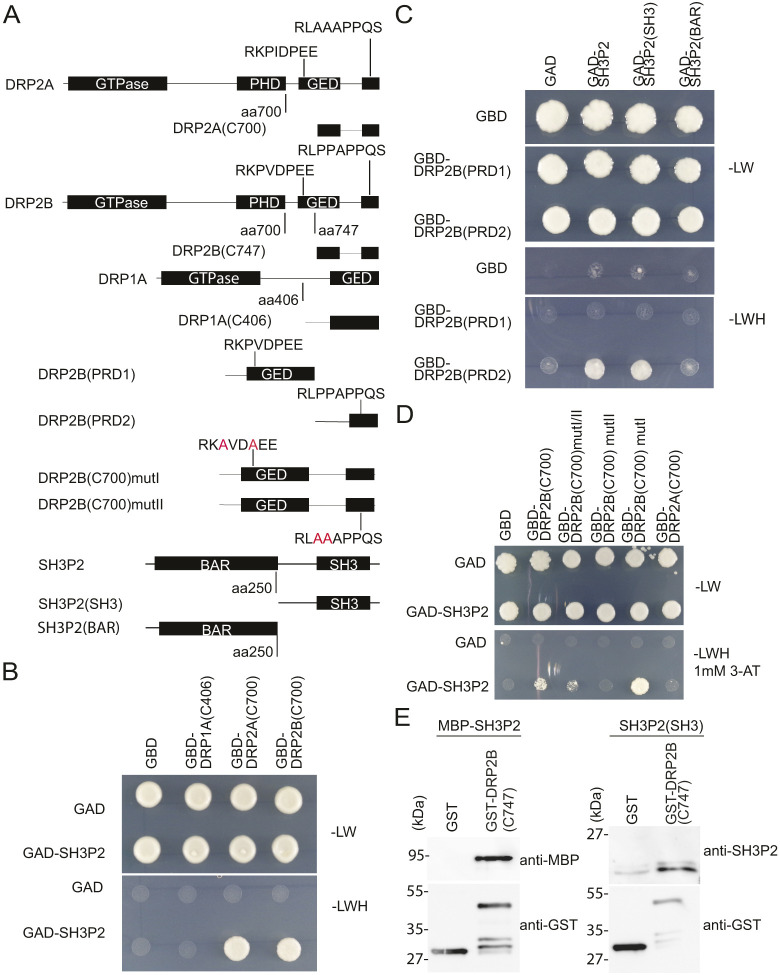
**Interaction of SH3P2 and DRP2 proteins *in vitro*.** (A) Schematic presentation of full-length DRP1A, DRP2A, DRP2B and SH3P2, the truncated constructs DRP1A(406), DRP2A(C700), DRP2B(C747), DRP2B (C700)mutI and DRP2B(C700)mutII, SH3P2 (SH3) and SH3P2 (BAR). (B) YTH analyses of GAD–SH3P2 with GBD fusions of DRP2A(700) and DRP2B(747) truncated versions, and an empty vector GAD and truncated version of GBD–DRP1A(406) as negative controls. (C) YTH analyses of interaction between GAD–SH3P2, GAD–SH3P2(SH3) and GAD–SH3P2(BAR) with GBD fusions of truncated versions of DRP2B PRM1 and PRM2. (D) YTH analyses of interaction between GAD–SH3P2 and GBD fusions of DRP2B(700) with point mutations in PRM1 (mutI), PRM2 (mutII) or both (mutI/II). Yeast transformants were grown on medium lacking leucine and tryptophan (−LW) or leucine, tryptophan and histidine (−LWH) supplemented with 1 mM 3-amino-1,2,4-triazole (3-AT) to test their auxotrophic growth. Empty vectors, GAD and GBD, were used as negative controls. (E) *In vitro* binding assay of SH3P2 with DRP2B(C747). GST was used as a negative control. Bead-bound GST and GST-DRP2B(C747) were incubated with equal amounts of full-length MBP-SH3P2. After intensive washing, bead-bound materials were subjected to immunoblotting using anti-MBP and anti-GST antibodies. Images representative of three repeats.

Given that the recruitment profiles of SH3P2 and DRP2s indicate their involvement in the terminal stages of the CME event, we further investigated the spatiotemporal relation between these proteins. Taking into account that their recruitment is similar to that of mammalian dynamin and BAR-domain proteins (Taylor et al., 2011), we hypothesised that there would be similar interaction mechanisms between SH3P2 and DRP2s. Although mammalian dynamin interacts with other proteins through its PRD of 13 proline-rich motifs (PRMs) (Okamoto et al., 1997), DRP2s contain only two highly conserved PRMs localised at the beginning of GED domain and in the middle of PRD, respectively (DRP2A, PRM1, RKPIDPEE, and PRM2, RLPPAPPPTG; DRP2B, PRM1, RKPVDPEE, and PRM2, RLPPAPPQS) (Hong et al., 2003; Schmid and Frolov, 2011). The scheme shown in Fig. 4A represents the truncated versions of the DRP2A(C700), DRP2B(C700;C747), DRP1A(C406), DRP2B (PRD1 and PRD2) and SH3P2(C250) that were used in the further experiments, as well as the point mutations in the PRMs.

First, we tested the interaction between SH3P2 and two C-terminal portions of DRP2 proteins, both containing PRMs, using a yeast two-hybrid (YTH) assay. As a negative control, we used the C-terminal portion of DRP1A protein, a member of DRP subfamily 1 that lacks both PH domain and PRD. Our results showed that the C-terminal portion of DRP2A and DRP2B, which contain both PRMs, were able to interact with SH3P2 in the YTH assay (Fig. 4B; Fig. S4A). As expected, we could not detect any interaction between SH3P2 and DRP1A. Together, these data suggest that SH3P2 interacts with the C-terminus of DRP2 and this interaction could be mediated through the PRMs in DRP2A and DRP2B. Next, we determined the importance of the DRP2 PRMs for the interaction with the SH3 domain of SH3P2. Minimal domain analyses using a YTH assay showed that PRM1 cannot interact with SH3 domain of SH3P2 alone, whereas PRM2 showed interaction with both SH3P2 full-length protein and the C-terminal fragment containing the SH3 domain (Fig. 4C). The N-terminus of SH3P2 containing the BAR domain did not interact with either PRM. Moreover, our results show that PRM1 cannot interact with SH3 domain of SH3P2 alone (Fig. 4C). In contrast, the presence of the PRM2 motif, located in PRD, alone was sufficient for the protein–protein interaction (Fig. 4C; Fig. S4B,C).

We further designed point mutations in one or both PRMs and tested their interaction with SH3P2 using YTH assays. To reduce autoactivation of the GAL4-binding domain, the selection medium was supplemented with 3-amino-1, 2, 4-triazole (3-AT). When both PRMs were mutated, we did not detect any interaction between DRP2B and SH3P2. The presence of the PRM2 motif alone was sufficient for the protein–protein interaction. Mutating PRM2 prevented the interaction with SH3P2, whereas mutation in PRM1 did not (Fig. 4D). The direct interaction between SH3P2 and PRM2 of DRP2B was confirmed by *in vitro* pulldown assay, using GST–DRP2B(C747), full-length protein MBP–SH3P2 and untagged SH3P2(SH3) (Fig. 4E). In summary, we confirm that SH3P2 can interact with both members of the DRP2 subfamily and that only PRM2 of DRP2B is crucial for the interaction with SH3 domain of SH3P2.

**Fig. 5.**
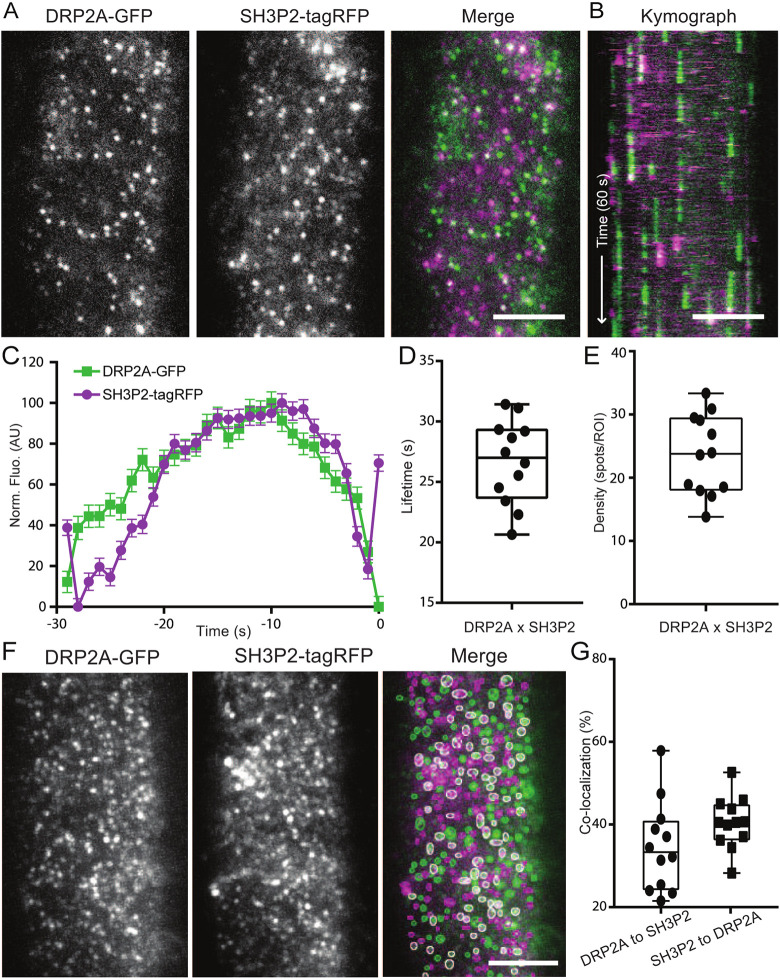
**DRP2A and SH3P2 colocalisation *in vivo*.** (A) TIRF-M images of a cell surface of root epidermal cell expressing *pDRP2A::DRP2A-GFP* and *pSH3P2::SH3P2-tagRFP*. (B) Representative kymograph of DRP2A and SH3P2 lifetimes on the PM. Arrow represents the time direction and timespan of 60 s. (C–E) Data from twelve independent experiments were combined to generate the (C) mean±s.e.m. recruitment profile of SH3P2-positive DRP2A foci; (D) mean±s.e.m. lifetime of CME events, 26.68±0.5 s; and (E) mean density of CME events, 23.63 spots per ROI. *n*=12 cells from independent roots, 16,916 tracks. (F) Representative image of colocalisation analysis of SH3P2 and DRP2A foci. The circled areas indicate detected spots (green and magenta circles for GFP and tagRFP, respectively). White circles represent detections in both channels. (G) Quantification of colocalised spots. 34.58% of DRP2A was colocalised to SH3P2, and 40.41% of SH3P2 was colocalised to DRP2A. *n*=12 cells from independent roots. For box plots in D, E and G, the box represents the 25–75th percentiles, and the median is indicated. The whiskers show the maximum to minimum range. AU, arbitrary units. Scale bars: 5 µm (A,B,F).

To further investigate the relevance of our *in vitro* observations for CME, we performed the following *in vivo* experiments. We generated plant lines containing SH3P2–tagRFP and DRP2A–GFP markers to study their dynamics and colocalisation *in vivo* (Fig. 5A,B). We were able to detect foci that were spatiotemporally positive in both channels and the average lifetime of these events was 26.6±0.14 s (mean±s.e.m.) (Fig. 5C–E). This lifetime is almost half that of the lifetime of DRP2A and SH3P2 with CME marker (∼46 s and ∼41 s, respectively) (Figs 1D and 2D), which reflects their arrival during the late stage of CME. The fluorescent profiles of DRP2A and SH3P2 suggest a simultaneous peak of their arrival, although DRP2A seems to arrive slightly earlier than SH3P2 (Fig. 5C). The average density of these events was 23.63 spots per ROI, which was lower than the foci density of DRP2A and SH3P2 with CLC2 (∼40 and ∼44 spots per ROI) (Figs 5E, 1E and 2E). We further checked the percentage of colocalised SH3P2–tagRFP and DRP2A–GFP foci (Fig. 5F) and found that a frequency of 34.6% for SH3P2–tagRFP and DRP2A–GFP, whereas 40.41% of DRP2A–GFP was colocalised with SH3P2–tagRFP at a given time point (Fig. 5G). These data suggest the existence of CME events that have either DRP2A or SH3P2 separately. However, for events that have both SH3P2 and DRP2A arriving at the PM together it is possible that they do function together, similar to what is seen for mammalian Endo2/Amph1 and dynamin.

**Fig. 6.**
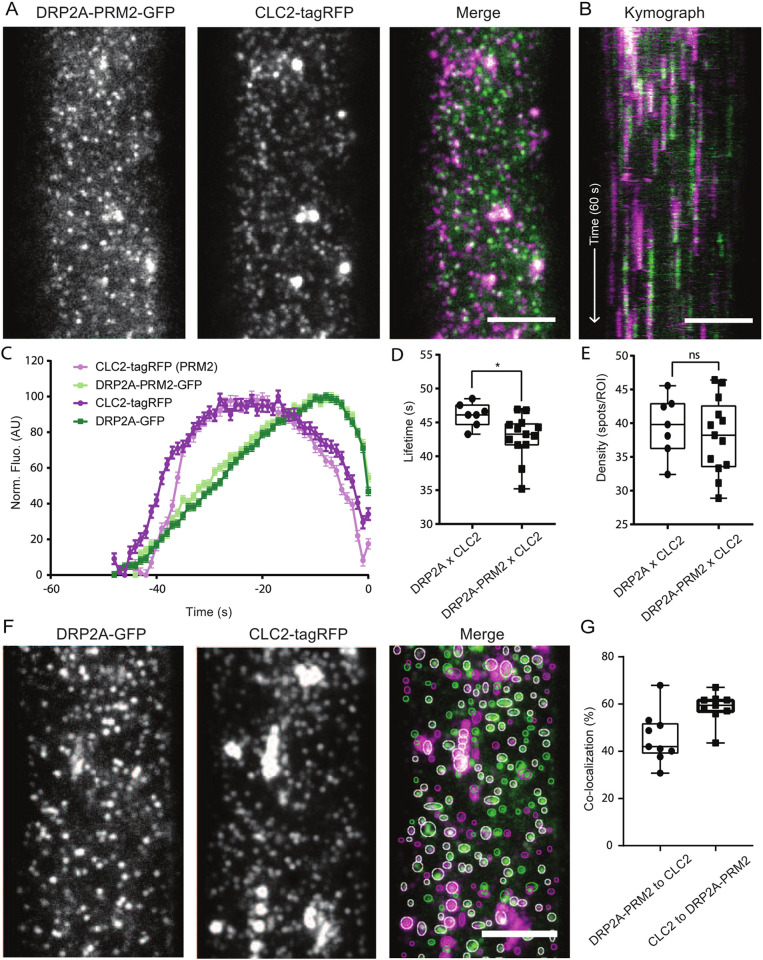
**PRM2 is important for interaction with SH3P2 *in vitro*, but not *in vivo*.** (A) TIRF-M images of a cell surface of root epidermal cell expressing *pDRP2A::DRP2A-PRM2-GFP* and *pRPS5A ::CLC2-tagRFP* (*drp2a-1−/−*). (B) Representative kymograph of DRP2A-PRM2 and CLC2 lifetimes on the PM. Arrow represents the time direction and timespan of 60 s. (C–E) Data from nine independent experiments for DRP2A-PRM2×CLC2 were combined and compared to DRP2A×CLC2 from Fig. 1, to generate the (C) mean±s.e.m. recruitment profile of DRP2A and DRP2A-PRM2 foci; (D) mean±s.e.m. lifetime of CME events, 43.72±0.18 s; and (E) mean density of CME events, 40.05 spots per ROI. DRP2A-PRM2×CLC2, *n*=9 cells from independent roots, 29,407 tracks. *P<0.05; ns, not significant (*P*>0.05) (unpaired two-tailed *t*-test to compare to control). (F) Representative TIRF-M image of colocalisation analysis of DRP2A-GFP and CLC2-tagRFP *(drp2a-1−*/*−)* foci. The circled areas indicate detected spots (green and magenta circles for GFP and tagRFP, respectively). White circles represent detections in both channels. (G) Quantification of colocalised spots. 45.81% (mean) of DRP2A was colocalised to CLC2, and 58.5% of CLC2 was colocalised to DRP2A. *n*=9 cells from independent roots. For box plots in D, E and G, the box represents the 25–75th percentiles, and the median is indicated. The whiskers show the maximum to minimum range. AU, arbitrary units. Scale bars: 5 µm (A,B,F).

To complement these *in vitro* interaction experiments with mutated versions of the PRM2s*,* we generated plant CLC2–tagRFP lines containing DRP2A–GFP with point mutations in the PRM2 (RL**AA**APP**QS**, bold indicates mutated residues) (Fig. 4A). We analysed the dynamics of DRP2A-PRM2–GFP×CLC2-tagRFP plants compared to wild-type (WT) DRP2A–GFP×CLC2–tagRFP plants using TIRF-M (Fig. 6A,B). The mean±s.e.m. lifetime of DRP2A-PRM2–GFP×CLC2–tagRFP was reduced by 3 s (43.72±0.1 s) compared to the control (46.12±0.6 s), but the foci density remained the same (Fig. 6C–E). Despite the presence of point mutations in this interaction site, ∼60% of CLC2 foci colocalised with DRP2A–PRM2 with dynamics of both proteins similar to the WT DRP2A (Fig. 6F,G). Additionally, we generated a similar mutation in PRM2 of DRP2B (RL**AA**APPQS). No significant change was detected in either the lifetime or foci density of the DRP2B-PRM2 mutant compared to WT DRP2B, showing that DRP2B was not affected by the PRM2 mutation (Fig. S5). Overall, although we detected a reduced lifetime of DRP2A-PRM2–CLC2 foci, these proteins had a dynamic behaviour at the PM, indicating that despite this, mutated DRP2s were efficiently recruited to the site of CCV formation.

Additionally, we tested CLC2 dynamics in case of overexpression of the SH3P2 SH3 domain without the membrane-binding domain. The abundance of the SH3 domain in the cytosol would be expected to sequester interacting proteins, like DRP2s, to prohibit them from recruitment to the PM, causing impairment of CME (Gad et al., 2000; Szaszák et al., 2002). To test this, we generated a line with a CLC2–GFP marker and overexpressed the SH3P2 SH3 domain tagged with mCherry marker or free mCherry as a control (Fig. S6A–D). Analysis of CLC2 foci dynamics on PM showed no difference in either lifetime or density (Fig. S6E–G).

To conclude, the PRM of DRP2s are involved in protein–protein interaction with SH3P2 *in vitro*; however, mutation in this motif did not significantly affect endocytosis or DRP2 dynamics *in vivo*, revealing no functional consequence of the *in vitro* interaction.

### *sh3p123* shows normal DRP2A, CLC2 and TPLATE dynamics

In order to further investigate the role of SH3P proteins within plant CME, we used a new mutant of all three proteins of the SH3P family. It was generated by combining CRISPR-Cas technique for SH3P1 and SH3P2 and T-DNA insertion line of SH3P3 (Adamowski et al., 2024). The *sh3p123* triple mutant has been reported to have defects in plant growth and development, as well as seed germination. These phenotypes are more pronounced than those reported for various combinations of T-DNA alleles (Ahn et al., 2017; Nagel et al., 2017). By contrast, the SH3P2 RNA interference (RNAi) line displays severe defects in seedling development, showing the importance of SH3P2 alone for plant development (Zhuang et al., 2013).

We used this *sh3p123* triple mutant line to investigate the potential impairment of CME and defects in CME marker and cargo dynamics. Prior to the visualisation of CME marker in this line, we assessed the general membrane uptake of PM in root epidermal cells by measuring the uptake of the non-permeable membrane dye FM4-46 (Bolte et al., 2004; Jelínková et al., 2019). This assay allows testing membrane internalisation through analysis of formation of intracellular vesicles, stained with FM4-64. We observed a significant reduction of membrane uptake in root cells of *sh3p123* triple mutant compared to that seen in the WT, *sh3p12* double and *sh3p3* single mutants, which all exhibited a normal rate of membrane internalisation (Fig. S7A,B). This is a significant impairment of PM internalisation; however, this does not directly represent the rate of CME in plant cells. Therefore, we further tested whether mutation of SH3P proteins influences the recruitment of DRP2A to the site of CCV formation.

**Fig. 7.**
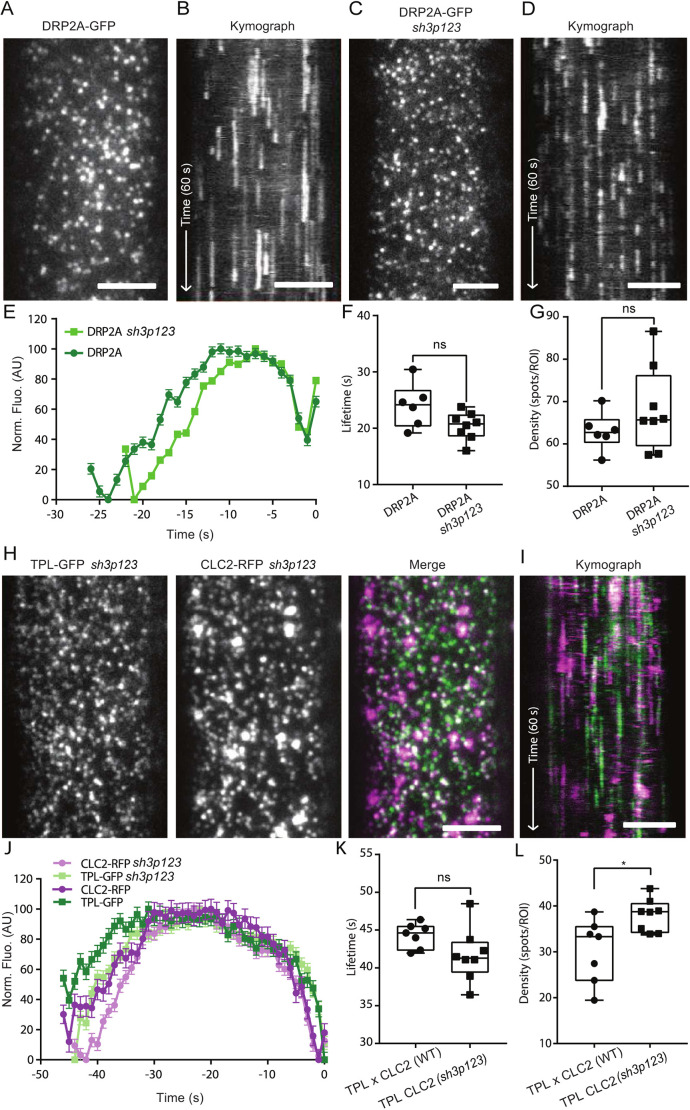
**CME markers have a normal lifetime in *sh3p123* triple mutant.** (A) TIRF-M images of a cell surface of root epidermal cell expressing *pDRP2A::DRP2A-GFP*. (B) Representative kymograph of DRP2A lifetimes on the PM. Arrow represents the time direction and timespan of 60 s. (C) TIRF-M images of a cell surface of root epidermal cell expressing DRP2A in *sh3p123* background. (D) Representative kymograph of DRP2A in *sh3p123* lifetimes on the PM. Arrow represents the time direction and timespan of 60 s. (E–G) Data from six independent experiments for DRP2A and eight independent experiments for DRP2A in *sh3p123* were combined to generate the (E) mean±s.e.m. recruitment profile of DRP2A foci; (F) mean±s.e.m. lifetime of CME events (DRP2A, 24.45±0.1 s; DRP2A *sh3p123* 20.1±0.08 s); and (G) mean density of CME events (DRP2A, 62.97 spots per ROI; DRP2A *sh3p123*, 68.24 spots per ROI). DRP2A, *n*=6 cells from independent roots, 41,473 tracks; DRP2A *sh3p123*, *n*=8 cells from independent roots, 77,361 tracks. ns, not significant (*P*>0.05) (unpaired two-tailed *t*-test to compare to control). (H) TIRF-M images of a cell surface of root epidermal cell expressing *pLAT52p::TPLATE-GFP* and *pRPS5A::CLC2-tagRFP (sh3p123).* (I) Representative kymograph of TPL×CLC2 in *sh3p123* background lifetimes on the PM. Arrow represents the time direction and the length of 60 s. (J–L) Data from seven independent experiments for TPL×CLC2 (Col-0) and eight independent experiments for TPL×CLC2 *(sh3p123)* were combined to generate the (J) mean±s.e.m. recruitment profile of TPL×CLC2 (Col-0) and TPL×CLC2 *(sh3p123)* foci; (K) mean±s.e.m. lifetime of CME events [TPL×CLC2 (Col-0), 44.2±0.3 s; TPL×CLC2 *(sh3p123),* 41.54±0.2 s]; and (L) mean density of CME events [TPL×CLC2 (Col-0), 30.29 spots per ROI; TPL×CLC2 *(sh3p123),* 38.02 spots per ROI]. TPL×CLC2 (Col-0), *n*=7 cells from independent roots, 16,367 tracks; TPL×CLC2 *(sh3p123)*, *n*=8 cells from independent roots, 24,217 tracks. *P<0.0155; ns, not significant (*P*>0.05) (unpaired two-tailed *t*-test to compare to control). For box plots in F, G, K and L, the box represents the 25–75th percentiles, and the median is indicated. The whiskers show the maximum to minimum range. AU, arbitrary units. Scale bars: 5 µm (A–D,H,I).

To do so we studied the dynamics of DRP2A–GFP in the *sh3p123* triple mutant background compared to the control DRP2A–GFP (Fig. 7A–D). Quantitative analysis of DRP2A lifetime persistence revealed that there was no significant difference between control and mutant plants (Fig. 7E,F). Also, no change in density of DRP2A foci was detected (Fig. 7G). These data contradict the hypothesis that SH3P proteins are crucial for the recruitment of DRP2s to the site of endocytosis, as DRP2A was still dynamically appearing and disappearing on the PM. To further investigate whether the *sh3p123* triple mutation influences the dynamics of other CME markers, we analysed the lifetime of total CLC2 events on the PM. Our findings are in line with previously shown data, where the CLC2 density in the *sh3p123* background had no difference to the control (Adamowski et al., 2024). We measured the CLC2–GFP dynamics in *sh3p123* background compared to WT (Fig. S7C–F) and saw a 1.7 s increase in lifetime in CLC2 for *sh3p123* plants; however, there was no difference in CLC2 density in the mutant (Fig. S7G–I). As the total observed clathrin foci at the PM also represent unsuccessful and aborted CME events, we used a plant line that, additionally to CLC2, expresses a fluorescently tagged member of TPLATE complex, TPL–GFP. Using TPL×CLC2 in the departure assay allowed us to analyse bona fide CME events in the *sh3p123* triple mutation (Fig. 7H,I). We visualise the dynamics of TPL×CLC2 positive foci and compared it to the control line (Fig. 7J). Quantitative analysis of the recruitment profiles of TPL and CLC2 positive events revealed no significant difference between control and mutant (Fig. 7J,K). The density of TPL×CLC2 was only slightly increased in the *sh3p123* mutant background compared to the control (Fig. 7L). In summary, the impaired membrane uptake observed in the *sh3p123* triple mutant was not caused by changes in functioning of CME components, like DRP2A, CLC or TPL.

### PIN2 recycling is not impaired in the *sh3p123* triple mutant

Previous reports have shown the SH3P2 binds ubiquitylated cargo and participates in vesicle trafficking (Nagel et al., 2017; Lam et al., 2001). Therefore, although SH3P2 might not have a major function during the process of CCV formation, it could potentially bind cargo immediately after the vesicle has been cut and clathrin coat has been disassembled, thus explaining why we detected no change in the dynamics of CME markers at the PM in *sh3p123* mutant.

**Fig. 8.**
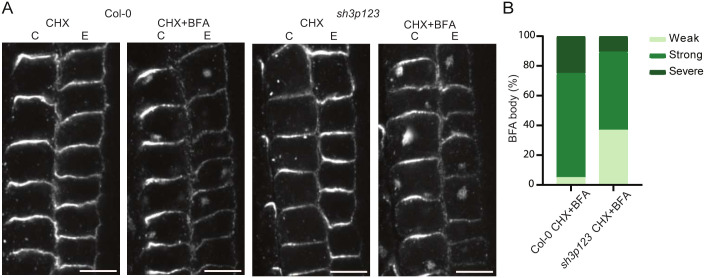
**Recycling of PIN2 proteins is normal in *sh3p123* triple mutant.** (A) Confocal images of immunolocalised PIN2 localisation in epidermis E and cortex C cells of *Arabidopsis* roots treated with CHX alone (50 µM for 1 h) or with BFA and CHX together (50 µM for 1 h) in Col-0 and *sh3p123* triple mutant. (B) A stacked column chart representing the percentage of roots, per genotype, distributed in three different categories (weak, strong or severe) based on the abundance of PIN2 BFA bodies (internalisation) formed in the cells after BFA treatment. Scale bars: 20 µm.

To test this hypothesis, we analysed the recycling of PIN2 in the *sh3p123* mutant, as this protein undergoes K63 ubiquitylation and constant recycling on PM through CME (Feraru et al., 2012; Leitner et al., 2012). Its recycling and polarity is dependent on proper functioning of CME machinery (Kitakura et al., 2011; Mravec et al., 2011). We used immunostaining of PIN2 in *A. thaliana* roots treated with brefeldin A (BFA), which blocks protein recycling at the Golgi (Naramoto et al., 2014). To observe the dynamics of only the PIN2 that was internalised through endocytosis and not synthesised *de novo*, we pre-treated samples with cycloheximide (CHX) (Schneider-Poetsch et al., 2010). An impairment in CME or trafficking of PIN proteins in *sh3p123* mutant would result in reduced amounts and/or size of BFA bodies. Surprisingly, analysis of PIN2 immunostaining showed only minor difference in amount and size of BFA formations compared to the control (Fig. 8A,B; Fig. S8A,B), suggesting that in *sh3p123* plants PIN2 has normal internalisation and recycling rates. Additionally, no influence of BFA, CHX or their combination compared to the control on the membrane abundances of PIN2 or membrane dynamics of CLC2 and DRP2A tagged with GFP was observed (Fig. S8C–I).

These observations are puzzling, as despite the severe phenotype and reduced levels of membrane internalisation observed with FM4-64, CME rates and cargo internalisation in *sh3p123* mutant plants are not significantly affected. This raises more questions about endocytosis in plants and function of SH3P proteins at the end of CCV formation.

## DISCUSSION

The scission machinery of CCVs is arguably one of the most important protein complexes in the CME mechanism. Although the scission process is well-characterised in mammalian and yeast systems, there are significant gaps in our understanding of how CCVs are released from the PM of the plant. Members of the DRP2 family, DRP2A and DRP2B, play a crucial role in plant growth and development (Backues et al., 2010). They have been hypothesised to do so through performing a similar scission function to their dynamin homologue in mammalian systems. Although some studies have shown colocalisation of DRP2s with other CME markers on the PM, it has not yet been clear when and how they arrive at the site of CCV formation. In this study, we aimed to gain insight into the composition of the potential scission machinery and its recruitment mechanism during plant CME. Specifically, we wanted to determine when DRP2s are recruited during the process of CCV formation and whether SH3Ps play an important role in this recruitment.

Using high-resolution live imaging of DRP2s and SH3P2 together with CLC2 on the PM of root epidermal cells allowed us to study the dynamics of these proteins in the scope of CCV formation, maturation and removal from the membrane. We found that both DRP2s, as well as SH3P2, arrive at a time that is close to the time of departure of CCV in the majority of endocytic events. Not only do they arrive and colocalise during endocytosis, but they interact as confirmed by YTH and pulldown assays. Using an *in vitro* approach, we established the specific motif within DRP2s that interacted with SH3 domain of SH3P2. Moreover, our *in vitro* data show that SH3P2 can deform membranes. These results support previously published data for DRP2s and SH3P2, and are also in line with the recruitment dynamics and biochemical properties of its mammalian and yeast homologues. However, our *in vivo* experiments with DRP2-PRM2 mutants, which could not interact with SH3P2 in YTH assays, showed no difference in DRP2-PRM2 nor CLC2 dynamics. What could be an explanation for this phenomenon? Closer analysis of recruitment profiles and colocalisation of DRP2A and SH3P2 suggests, that (1) recruitment of DRP2A starts before the recruitment of SH3P2, and (2) a significant amount of DRP2A does not colocalise with SH3P2, suggesting that DRP2s can be recruited through other mechanisms that do not involve SH3P2. Potentially, other members of DRP family are able to interact and recruit each other, like DRP1s or DRP2B, as GTPases can polymerase through their GED domain rather than the PRD, and therefore recruit DRP2s with a mutated PRM2 (Chappie et al., 2010; Fujimoto et al., 2008).

Next, we further investigated the functions of SH3P2 in CME. We used a recently generated triple mutant line that has a reduction of SH3P protein levels to study endocytosis rates (Adamowski et al., 2024). Contradictory to the decrease in overall membrane internalisation in *sh3p123*, we observed normal dynamics and density of DRP2A, CLC2 as well as TPLATE CME markers. The analysis of PIN2 dynamics in *sh3p123* mutant also did not show any changes compared to the control, indicating normal cargo recruitment and internalisation during CME. It needs to be mentioned that FM uptake has not always been a reliable representation of endocytosis in plants. An earlier study has shown that although plants with a mutation in CHC have significantly reduced FM uptake, the cargo recycling in these plants is not affected (Kitakura et al., 2011). These unexpected results raise further questions about the function of SH3P2 in CME and the unexplained behaviour of *sh3p123*, and show the need for further studies of these proteins.

Although our research was focused on the role of DRP2s and SH3P2 in plant CME, these proteins can be potentially involved in other types of endocytosis that are independent of clathrin. However, our knowledge about plant clathrin-independent endocytosis (CIE) and proteins mediating it is very limited. Interestingly, the N-BAR domain of SH3P2 is homologous to that of the mammalian endophilin which, among its functions in CME, mediates a dynamin-dependant CIE pathway called fast endophilin-mediated endocytosis (FEME) (Ahn et al., 2017; Casamento and Boucrot, 2020). The FEME pathway serves as a main internalisation road for fast (5–10 s) cargo internalisation for proteins like the β1-adrenergic receptor (β1AR) and cytokine receptors (Boucrot et al., 2015; Ferreira et al., 2021). In plants, partially characterised CIE suggests the involvement of Flot1, a homologue of mammalian flotillin that mediates membrane bending during CIE, which is a dynamin-dependant process (Glebov et al., 2006; Li et al., 2012). Taking into account that CLC2 foci have a higher colocalisation percentage with SH3P2 and DRP2s than their colocalisation percentage with clathrin, this gives an indication of clathrin-independent DRP2-dependant endocytosis pathway in plants. Moreover, the ability of SH3P2 to deform membranes, partial colocalisation of SH3P2 with clathrin foci and the lack of effects of mutation of SH3Ps on CME dynamics, mean it can be speculated that SH3P2 plays a role in the CIE mechanism, which remains much less characterised in plants (De Angelis et al., 2023; Li et al., 2012; Yu et al., 2020); nonetheless, more research needs to be dedicated to unravel the exact mechanism of such potential pathway and the cargoes that can be internalised through it.

In conclusion, our study provides detailed insights into the dynamics and interaction between DRP2 and SH3P2 proteins during CME in plants. We found that although DRP2s and SH3P2 arrive at the end of CCV formation and show specific interaction *in vitro*, DRP2 is likely to be recruited through other, yet-to-be-uncovered, mechanisms rather than exclusively through SH3P2. These results provide further evidence that although plants possess many homologues of proteins known to act in the mammalian and yeast CME system, plant CME functions in a rather unique and often unpredictable way.

## MATERIALS AND METHODS

### Plant material and growth conditions

All *Arabidopsis thaliana* mutants and transgenic lines used are in Columbia-0 (Col-0) background. *Arabidopsis thaliana* accession codes for genes used in this study are: DRP2A (AT1G10290), DRP2B (AT1G59610), SH3P2 (AT4G34660.1), CLC2 (AT2G40060), TPLATE (AT3G01780), PIN1 (AT1G73590), PIN2 (AT5G57090). Transgenic *Arabidopsis thaliana* plants used in this study were: *drp2-1a−*/*−, drp2b-2−/−, drp2a-1−/−;drp2b-2+/−,* pDRP2A::DRP2A-GFP *(drp2a-1−/−),* pDRP2A::DRP2A-GFP *(drp2a-1−/−;drp2b-2−/−),* pDRP2A::DRP2A-GFP×pRPS5A::CLC2-tagRFP *(drp2a-1−/−)*, pDRP2A::DRP2A-PRM2-GFP×pRPS5A::CLC2-tagRFP *(drp2a-1−/−)*, pDRP2B::DRP2B-GFP×pRPS5A::CLC2-tagRFP *(drp2b-2−/−)*, pDRP2B::DRP2B-PRM2-GFP×pRPS5A::CLC2-tagRFP *(drp2b-2−/−)*, pSH3P2::SH3P2-GFP×pRPS5A::CLC2-mOrange (Col-0), pDRP2A::DRP2A-GFP×pSH3P2::SH3P2-tagRFP (Col-0), *sh3p12, sh3p3, sh3p123,* pXVE:UBQ10::mCherry×pRPS5A::CLC2-GFP (Col-0), pXVE:UBQ10::SH3-mCherry×pRPS5A::CLC2-GFP (Col-0), pDRP2A::DRP2A-GFP *(sh3p123),* pSH3P2::SH3P2-GFP (Col-0), pSH3P2::SH3P2-GFP *(sh3p123),* pRPS5A::CLC2-GFP (Col-0), pRPS5A::CLC2-GFP *(sh3p123),* pLAT52p::TPLATE-GFP×pRPS5A::CLC2-tagRFP (Col-0), pLAT52p::TPLATE-GFP×pRPS5A::CLC2-tagRFP *(sh3p123)* (Adamowski et al., 2024; Backues et al., 2010; Gadeyne et al., 2014; Nagel et al., 2017; Smith et al., 2014)*. Arabidopsis thaliana* seeds were surface-sterilised with either chlorine gas or 99% ethanol for 10 min. Seeds were then plated onto AM+ medium plates [1/2-Murashige–Skoog with 1% (w/v) sucrose and 1% agar], stratified for 1 to 2 days in the dark at 4°C, and then transferred to the growth room (21°C, 16 h light, 8 h dark) and grown vertically for 5 or 7 days depending on the type of experiment for which they were required. Light sources used were Philips GreenPower LED production modules [in deep red (660 nm)/far red (720 nm)/blue (455 nm) combination, Philips], with a photon density of 140.4 μmol/m^2^/s±3%.

### Cloning

Constructs for generating plant fluorescent reporter lines were generated using the Gate Way protocol (Thermo Fisher Scientific). In brief, promoter sequences were amplified from gDNA generated from Col-0 plants and put in the p41r entry vector. Gene sequences were cloned using cDNA generated from Col-0 plants into the pDONR221 entry vector. Fluorescent tags (GFP or mCherry) were cloned into the p23 entry vector. The final plasmids were assembled using p41r, pDONR221 and p23 entry vectors and destination vectors with desired antibiotic resistance. *Agrobacterium tumefaciens* strain pGV3101 was used to deliver destination constructs into corresponding plants by floral dip.

Cloning of GAD–SH3P2, MBP–SH3P2(FL) and GST–SH3P2(SH3) was undertaken as previously described in Nagel et al. (2017). For GAD–SH3P2(SH3) and GAD–SH3P2(BAR), the fragments of SH3P2 were amplified with primers MN176-MS12 (SH3P2(SH3)) and MS11-MN177 (SH3P2(BAR)) and cloned between the EcoRI and XhoI and EcoRI and BamHI restriction sites of pGADT7 (Clontech), respectively. To obtain GBD–DRP1A(C406), GBD–DRP2A(C700) and GBD–DRP2B(C700), the fragments were amplified from *Arabidopsis* cDNA with the primer pairs MN349-MN226 (DRP1A(C406)), MN316-MN321 (DRP2A(C700)), and MN317-MN315 (DRP2B(C700)) and cloned between the NdeI and XhoI (DRP1A(C406)) and NdeI and EcoRI(DRP2A/B(C700)) restriction sites of pGBKT7. Mutated versions were amplified from DRP2B pDONR221 construct used as template using primer pairs MN317-MN315 and cloned between NdeI and EcoRI (DRP2A/B(C700)) restriction sites of pGBKT7. GBD–DRP2B(PRD1) and GBD–DRP2B(PRD2) were amplified with primers MN317-MN350 (DRP2B(PRD1)) and MN351-MN315 DRP2B(PRD2) and cloned between NdeI and EcoRI restriction sites of pGBKT7. GST–DRP2B(C474) was amplified by primer pairs MN256-MN255 and cloned between the EcoRI and XhoI restriction sites of pGEX-6p-1.

Constructs for protein overexpression in bacteria were generated using a Gibson Assembly protocol. A GBlock gene with overhangs for the destination vector sequence was codon-optimised for *Escherichia coli* expression using the IDT service and then inserted into a pTB146 destination vector. A His-SUMO tag was located on the N-terminus of the expression construct. A list of primers used for cloning is listed in Table S1.

### FM uptake assay, TIRF-M imaging and analysis

A Zeiss inverted LSM-800 confocal microscope equipped with Airyscan and 40× water immersion objected was used to examine the FM4-64 uptake in 7-day-old seedlings. Seedlings were incubated for 15 min at room temperature with 2 µM FM4-64 in AM+ medium [1/2-Murashige–Skoog with 1% (w/v) sucrose], washed three times in AM+ medium, and imaged and analysed, as described previously (Johnson et al., 2020). For TIRF-M experiments an Olympus IX83 inverted microscope equipped with a Cell^TIRF module using an OLYMPUS Uapo N 100×/1.49 Oil TIRF objective was used. Cut roots were mounted on the slide in AM+ medium and covered with precision cover glasses (refractive index 1.5H). Root epidermal cells were imaged and data was analysed as described previously (Johnson et al., 2020). Co-localisation of proteins *in vivo* was done using Spots colocalisation (ComDet v.0.5.5) plugin for ImageJ (https://github.com/UU-cellbiology/ComDet).

### YTH assay

GAD and GBD fusion constructs were co-transformed into yeast strain Y8800 (based on PJ69-4; MATa, leu2-3,112 trp1- 901 his3-200 ura3-52 gal4Δ gal80Δ GAL2-ADE2 LYS2::GAL1- HIS3 MET2::GAL7-lacZ cyh2R). Transformants were selected after 3 day on synthetic complete (SC) medium [yeast nitrogen base without amino acids and with 6.7 g/l ammonium sulfate, 2% (w/v) agar, 2% (w/v) glucose and 1× amino acid dropout mix (Sigma-Aldrich/Merck, Y0750)] lacking leucine and tryptophan (−LW) at 30°C. To examine reporter gene expression, transformants were grown on solid SC medium lacking leucine, tryptophan and histidine (−LWH) supplemented with 5 mM 3-amino-1,2,4-triazole for 2 days at 30°C. Yeast total proteins were extracted as described previously (Nagel et al., 2017), and expression of constructs was analysed by immunoblotting using anti-GBD and anti-HA (GAD) antibodies [anti-GAL4(DBD) (mouse) (RK5C1), monoclonal, Santa Cruz Biotechnology, sc-510, 1:3000; anti-HA (rat) (3F10), monoclonal, Roche, 11867423001, 1:2000].

### Protein purification and *in vitro* binding assays

GST- or MBP-fused proteins were expressed in the *Escherichia coli* Rosetta (DE3), Rosetta-gami 2, or Rosetta-gami B strains (all from Merck Millipore) and purified using Pierce glutathione magnetic beads (Thermo Fisher Scientific), or Amylose Resin (New England Biolabs), depending on the tag of the fusion protein. For SH3P2(SH3) the GST tag was removed with PreScission Protease (Cytiva). For *in vitro* binding assays, Pierce glutathione magnetic beads saturated with 80 pmol of the GST fusion protein GST–DRP2B(C747) were incubated with an equimolar amount of MBP–SH3P2 or untagged SH3P2(SH3) in 400 μl of cold buffer (50 mM Tris-HCl pH 7.5, 150 mM NaCl, 10 mM MgCl_2_ and 0.05% Tween-20) under rotation at 4°C. The beads were then washed four times with cold buffer, proteins were eluted with 40 mM glutathione. Bead-bound materials were subjected to SDS/PAGE and analysed by immunoblotting. Full images of blots shown in the paper are provided in Fig. S9. The following antibodies were used in the experiment in the mentioned dilutions. Primary antibodies were: anti-GST (rabbit), polyclonal, generated for this study by Eurogentech, 1:1000; anti-MBP (mouse), monoclonal, New England Biolabs, E8032L, 1:10,000; anti-GAL4(DBD) (mouse) (RK5C1), monoclonal, Santa Cruz Biotechnology, sc-510, 1:3000; anti-HA (rat) (3F10), monoclonal, Roche, 11867423001, 1:2000; and anti-SH3P2 (rabbit), polyclonal, generated for this study (Eurogentech), 1:1000. Secondary antibodies were: anti-rat-IgG conjugated to HRP (goat), Sigma-Aldrich, A9037, 1:5000; anti-mouse IgG conjugated to HRP (rabbit), Sigma-Aldrich, A9044, 1:80,000; and anti-rabbit IgG conjugated to HRP (goat), Sigma-Aldrich, A0545, 1:80,000.

### Purification of SH3P2 protein

SH3P2 was cloned into vector pTB146, with an N-terminal 6×His tag followed by SUMO fusion protein using Gibson assembly protocol (Thermo Fisher Scientific). Protein was expressed in *E. coli* BL21 cells, grown at 30°C (250 rpm) in LB medium supplemented with 100 µg/ml^−1^ ampicillin. Prior to expression the cell culture was cooled down to 4°C, and expression was induced at an optical density at 600 nm (OD600) of 0.8 with 1 mM IPTG. The protein was expressed overnight at 12°C and harvested by centrifugation (5000 ***g*** for 30 min at 4°C). The pellet was resuspended in lysis buffer (50 mM Tris-HCl pH 8.0, 400 mM NaCl, 25 mM KH_2_PO_4_ pH 8.0, 10% Glycerol, 5 mM EDTA, 5 mM DTT, 1% Triton X-100 and 1 mM PMSF) supplemented with EDTA-free protease inhibitor cocktail tablets (Thermo Fisher Scientific) and 1 mg/ml^−1^ DNase I (NEB) and 1 mg/ml^−1^ lysozyme (Thermo Fisher Scientific). Cells were lysed by sonication using a Q700 Sonicator equipped with a probe of 12.7 mm diameter, which was immersed into the resuspended pellet. The suspension was kept on ice during sonication (Amplitude 25, 1 s ON and 4 s OFF for a total time of 10 min). Subsequently, cell debris was removed by centrifugation at 60,000 ***g*** for 1 h at 4°C. The clarified lysate was incubated with Ni-NTA resin for 1 h at 4°C. Then, the resin was washed with 20× CV washing buffer A (50 mM Tris-HCl pH 8 .0, 400 mM NaCl, 25 mM KH_2_PO_4_ pH 8.0, 10% glycerol, 5 mM EDTA, 5 mM DTT, 0.2% Triton X-100) and 40× CV washing buffer B (50 mM Tris-HCl pH 8.0, 400 mM NaCl, 25 mM KH_2_PO_4_ pH 8.0, 10% glycerol, 5 mM EDTA, 5 mM DTT and 20 mM imidazole). The fusion protein was eluted using elution buffer (50 mM Tris-HCl pH 8.0, 400 mM NaCl, 25 mM KH_2_PO_4_ pH 8.0, 10% glycerol, 5 mM EDTA, 5 mM DTT) containing increasing concentrations of imidazole (50–300 mM). The protein concentration was determined with a Bradford assay. The 6×His tagged protease UlpI was added in a 1:100 molar ratio, and the 6×His-SUMO tag was cleaved overnight at 4°C, accompanied by dialysis in dialysis buffer (50 mM Tris-HCl pH 8.0, 400 mM NaCl, 25 mM KH_2_PO_4_ pH 8.0, 10% glycerol, 5 mM EDTA and 5 mM DTT) with gentle stirring. To exchange SH3P2 into the final buffer (25 mM HEPES pH 7.5, 200 mM NaCl, 10% glycerol, 1 mM EDTA, 5 mM DTT and 25 mM KH_2_PO_4_ pH 8.0) PD10 columns (Cytiva) were used. To remove the cleaved tag and UlpI protease, SH3P2 was subjected to reverse affinity chromatography. The purity of the final protein was determined via SDS-PAGE and protein concentration was determined via NanoDrop and Bradford assays. Protein was flash-frozen in liquid nitrogen and stored at −80°C.

### LUV preparation

LUVs were prepared using a mixture of 1,2-dioleoyl-sn-glycero-3-phospho-(1′-rac-glycerol), 1,2-dioleoyl-sn-glycero-3-phospho-L-serine, 1,2-dioleoyl-sn-glycero-3-phosphate (at a ratio of 60:20:20 mol%) and 1,2-dioleoyl-sn-glycero-3-phospho-(1′-myo-inositol-4′,5′-bisphosphate) [PI(4,5)P_2_] (Avanti) at a ratio of DOPC (100, mol%), DOPC:DOPS (80:20, mol%), DOPC:DOPS:PA (80:18:2, mol%) and DOPC:DOPS:PI(4,5)P_2_ (80:17.5:2,5mol%). Lipids were mixed in a glass vial at the desired ratio, blow-dried with filtered N_2_ to form a thin homogeneous film, and kept under vacuum for 2 to 3 h. Next, the lipid film was rehydrated in a swelling buffer (25 mM HEPES pH 7.5, 200 mM NaCl, 25 mM KH_2_PO_4_ pH 8.0) for 10 min at room temperature. The total lipid concentration was 2 mM. The mixture was vortexed rigorously, and the resulting dispersion of multilamellar vesicles was repeatedly freeze thawed (five to six times) in liquid nitrogen. The mixture was extruded through a polycarbonate membrane with a pore size 400 nm (LiposoFast Liposome Factory). LUVs were stored at 4°C and used within 4 days.

### Sedimentation assay

To assess membrane-binding capacity of SH3P2, a pelleting assay was used. In brief, LUVs were prepared as described above with swelling buffer containing 200 mM sucrose instead of NaCl. SH3P2 protein was cleaned from aggregates using ultracentrifugation at 100,000 ***g*** (TLA-100 rotor) for 20 min at 4°C. LUVs were incubated with the protein for 10 min at room temperature and spun down at 100,000 ***g*** (TLA-100 rotor) for 20 min at 4°C. Supernatant was separated and pellet was suspended in outside buffer (25 mM HEPES pH 7.5, 200 mM NaCl, 25 mM KH_2_PO_4_ pH 8.0). Samples were assessed SDS-PAGE. Images of gels were analysed using a GelAnalyzer 19.1 (www.gelanalyzer.com).

### Tubulation assay

To test membrane-bending activity of SH3P2, electron microscopy of LUVs incubated with 1 µM of SH3P2 was undertaken. The final concentration of LUVs was 0.5 mM. Protein with LUVs or LUVs alone were incubated for 10 min at room temperature. A total of 20 μl mix was incubated on glow-discharged carbon-coated copper EM grids (300 mesh, EMS). Filter paper was used to remove any excess solution. Grids were then negatively stained with 2% uranyl acetate aqueous solution for 1 min and observed under a Tecnai 12 transmission electron microscope operated at 120 kV (Thermo Fisher Scientific). Images were analysed using ImageJ.

### Mass photometer assay

To analyse the mass of individual protein molecules of SH3P2, a mass photometer assay was used. Coverslips were cleaned by sonication in mqH_2_O, isopropanol and again mqH_2_O for at least 5 min each. Protein was diluted to 50 nM, 75 nM and 100 nM final concentration in the final buffer (25 mM HEPES pH 7.5, 200 mM NaCl, 25 mM KH_2_PO_4_ pH 8.0). After adding the protein of interest, data was recorded at a framerate of 5 ms/frame and recorded for 1 min. Results were analysed using MP Discovery Software.

### Plant tissue immunostaining with BFA treatment

Whole-mount immunolocalisation was performed on 4-day-old seedlings of *Arabidopsis* following a previously published protocol (Sauer and Friml, 2010). In brief, the seedlings were pre-treated with control (DMSO) or 50 µM cycloheximide (CHX; Sigma-Aldrich) for 30 min, followed by a co-treatment with brefeldin A (BFA; Sigma-Aldrich) for 1 h with each at a concentration of 50 µM. Staining was undertaken with rabbit anti-PIN2 (produced and processed within our laboratory) diluted 1:1000 and CY3-conjugated anti-rabbit-IgG secondary antibody (Sigma, C2306) at 1:600. For confocal laser scanning microscopy, scans were taken using a Zeiss LSM800 microscope. The phenotype was scored based on the number of PIN2-containing BFA bodies formed in root cells. The categories classify roots on the basis of whether PIN2-containing BFA bodies were weakly, strongly or severely formed. The ‘severe’ category was assigned when all the imaged cells of a given root had at least one BFA body, whereas the ‘strong’ category was assigned to roots where most of the cells had BFA bodies, and the ‘weak’ category was assigned to roots that had very few BFA bodies. The experiment and analysis were performed in a masked manner, where the genotypes and the conditions were only revealed after the scoring was done twice.

### Ovule phenotyping

Immature siliques were removed from the flowering plants of different genotypes and placed on double-sided tape on microscopic slides. The siliques were opened along the replum using a hypodermic needle and immediately observed under a stereomicroscope (Olympus SZX16). In a masked experiment, the ovules were scored as aborted when they appeared as small, white, fist-like structures visibly different from fertilised and developed ovules.

## Supplementary Material



10.1242/joces.261720_sup1 Supplementary information
