# Supplementary material for: Role of the dynamin-related protein 2 family and SH3P2 in clathrin-mediated endocytosis in Arabidopsis thaliana
Source: J Cell Sci. 2024 May 2;137(8):jcs261720. doi: 10.1242/jcs.261720 (PMC11112126; doi:10.1242/jcs.261720)
Supplement: Supplementary information [file joces-137-261720-s1.pdf]

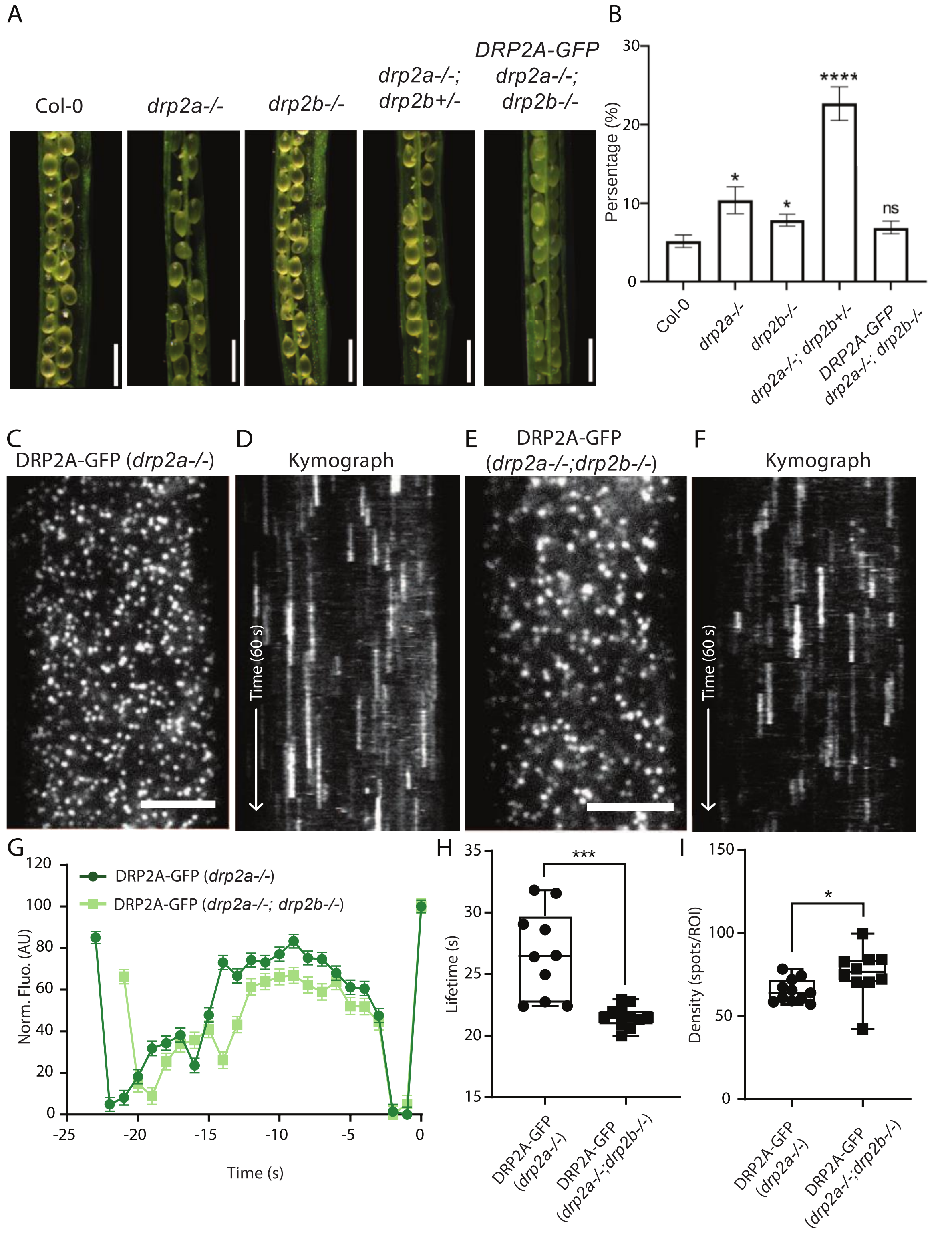

Fig. S1. DRP2A-GFP dynamics in *drp2a-/-drp2b-/-* background. (A). Ovule abortion phenotype assessment in Col-0, *drp2a-1-/-*, *drp2b-2-/-*, *drp2a-1-/-;drp2b-2+/-* and complemented *pDRP2A::DRP2A-GFP* in *drp2a-1-/-;drp2b-2-/-* plants. Representative images show open siliques of indicated genotypes. Scale bar, 1 mm. (B) Quantification of the percentage of ovule abortion phenotype is shown as mean of aborted ovules per silique ± SEM.

Journal of Cell Science • Supplementary information

Brown-Forsythe and Welch one-way ANOVA test with Dunnett's multiple comparison post hoc test to compare each genotype to the wild-type values. *, P value = 0.0332; ****, P value < 0.0001, ns – no statistical significance. Between 15 and 17 siliques, each containing 40 to 60 ovules per genotype were analysed. (C) TIRF-M image of a cell surface of root epidermal cell expressing *pDRP2A::DRP2A-GFP* in *drp2a-1-/-* background and (D) a representative kymograph of DRP2A lifetime on the PM. Scale bar: 5 µm. Arrow represents the time direction and the length of 60 sec. (E) TIRF-M image of a cell surface of root epidermal cell expressing *pDRP2A::DRP2A-GFP* in *drp2a-1-/-;drp2b-2-/-* background (scale bar: 5 µm) and (F) a representative kymograph of DRP2A lifetime on the PM. Arrow represents the time direction and the length of 60 sec. (G-I) Data from eleven independent experiments for DRP2A in *drp2a-1-/-* and ten independent experiments for DRP2A in *drp2a-1-/-;drp2b-2-/-* were combined to generate a (G) mean recruitment profile of DRP2A in *drp2a-1-/-* and DRP2A-GFP in *drp2a-1-/-;drp2b-2-/-* foci, (H) mean lifetime (DRP2A *drp2a-1-/-*, 26.65±1.13 s; DRP2A *drp2a-1-/-;drp2b-2-/-*, 21.55±0.2 s), and (I) mean density (DRP2A *drp2a-1-/-*, 65.23±2.1 spots ROI^{-1}; DRP2A-GFP *drp2a-1-/-;drp2b-2-/-*, 75.99±4.6 spots ROI^{-1}) of CME events. Plots indicate Mean±SEM DRP2A *drp2a-1-/-*, n=11 cells from independent roots, 130,535 tracks; DRP2A *drp2a-1-/-;drp2b-2-/-*, n=10 cells from independent roots, 88,646 tracks. Plot, Mean ± SEM, ***P < 0.0002; *P < 0.0432, t-test to compare to control.

Journal of Cell Science • Supplementary information

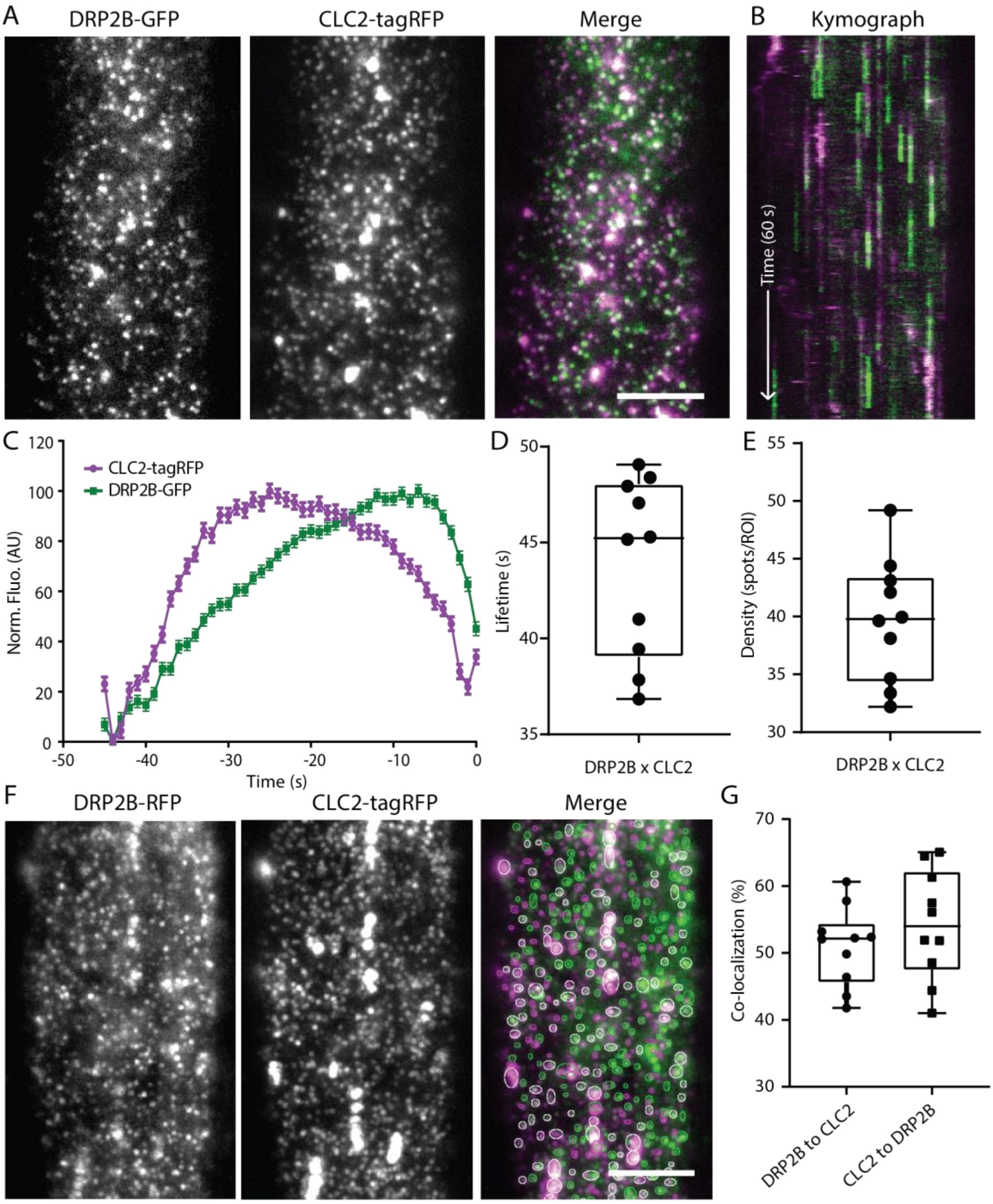

Fig. S2. Dynamics of DRP2B on the PM. (A) TIRF-M images of a cell surface of root epidermal cell expressing fluorescently tagged *pDRP2B::DRP2B-GFP* and *pRPS5A::CLC2-tagRFP* (*drp2b-2-/-*). Scale bar: 5 µm. (B) Representative kymograph of DRP2B and CLC2 lifetimes on the PM. Arrow represents the time direction and the length of 60 sec. (C-E) Data from ten independent experiments were combined to generate a (C) mean recruitment profile of DRP2B to the site of endocytosis, (D) mean lifetime 43.01±0.18 s, and (E) mean density 39.66 ±1.6 spots $ROI^{-1}$of CME events. Plots indicate Mean±SEM, n=10 cells from independent roots, 27,640 tracks. (F) Representative image of co-localisation analysis of DRP2B and CLC2 foci. Scale bar: 5 µm. (G) Quantification of co-localised spots. 50.99±1.8% of DRP2B were co-localised to CLC2, and 54.22±2.5% of CLC2 were co-localised to DRP2B.

Journal of Cell Science • Supplementary information

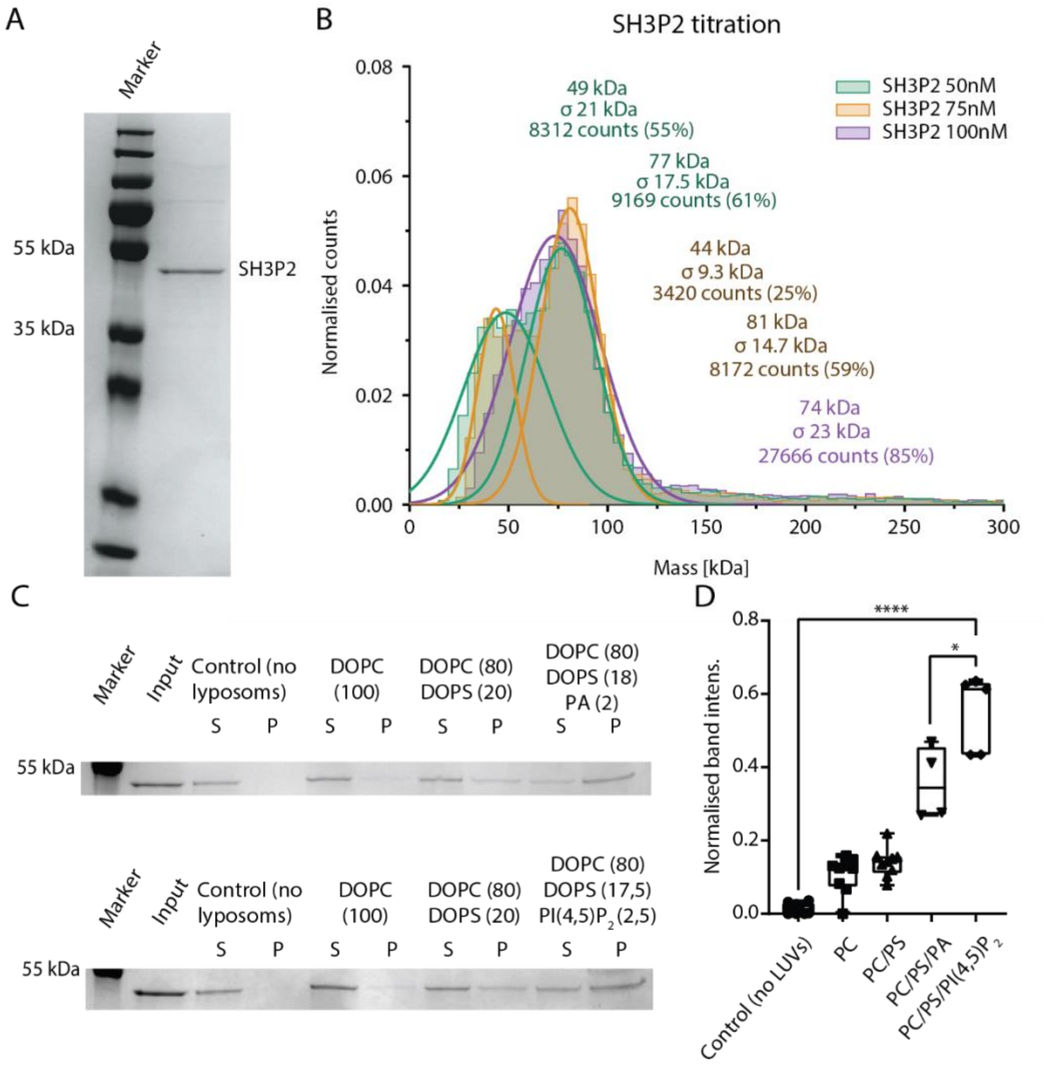

Fig. S3. SH3P2 characterization *in vitro*. (A) SDS-PAGE gel of the purified full-length (FL) SH3P2 protein. (B) Mass photometry (MS) analysis of SH3P2 protein in final concentrations of 50 nM, 75 nM, and 100 nM. The histogram shows the population distributions of purified FL SH3P2. Estimated molecular weights for the monomer and dimer and even counts are indicated on each graph. (C) Representative SDS-PAGE gel images of co-sedimentation assay testing lipid-binding capacity of SH3P2 to LUVs with different lipid composition: DOPC (100, mol%), DOPC:DOPS (80:20, mol%), DOPC:DOPS:PA (80:18:2, mol%), DOPC:DOPS: PI(4,5)P2 (80:17.5:2,5 mol%). S- supernatant; P-pellet. (D) Quantification of normalised Pellet band intensity of SH3P2 sedimentation assay, N repeats, control (no liposomes)=7, DOPC (100%, mol%)=7, DOPC:DOPS (80:20, mol%)=8, DOPC:DOPS:PA (80:18:2, mol%)=5, DOPC:DOPS: PI(4,5)P2 (80:17.5:2,5 mol%)=5. Plot, Mean ± SEM, ***P < 0.0002; *P < 0.0269, t-test to compare to control.

Journal of Cell Science • Supplementary information

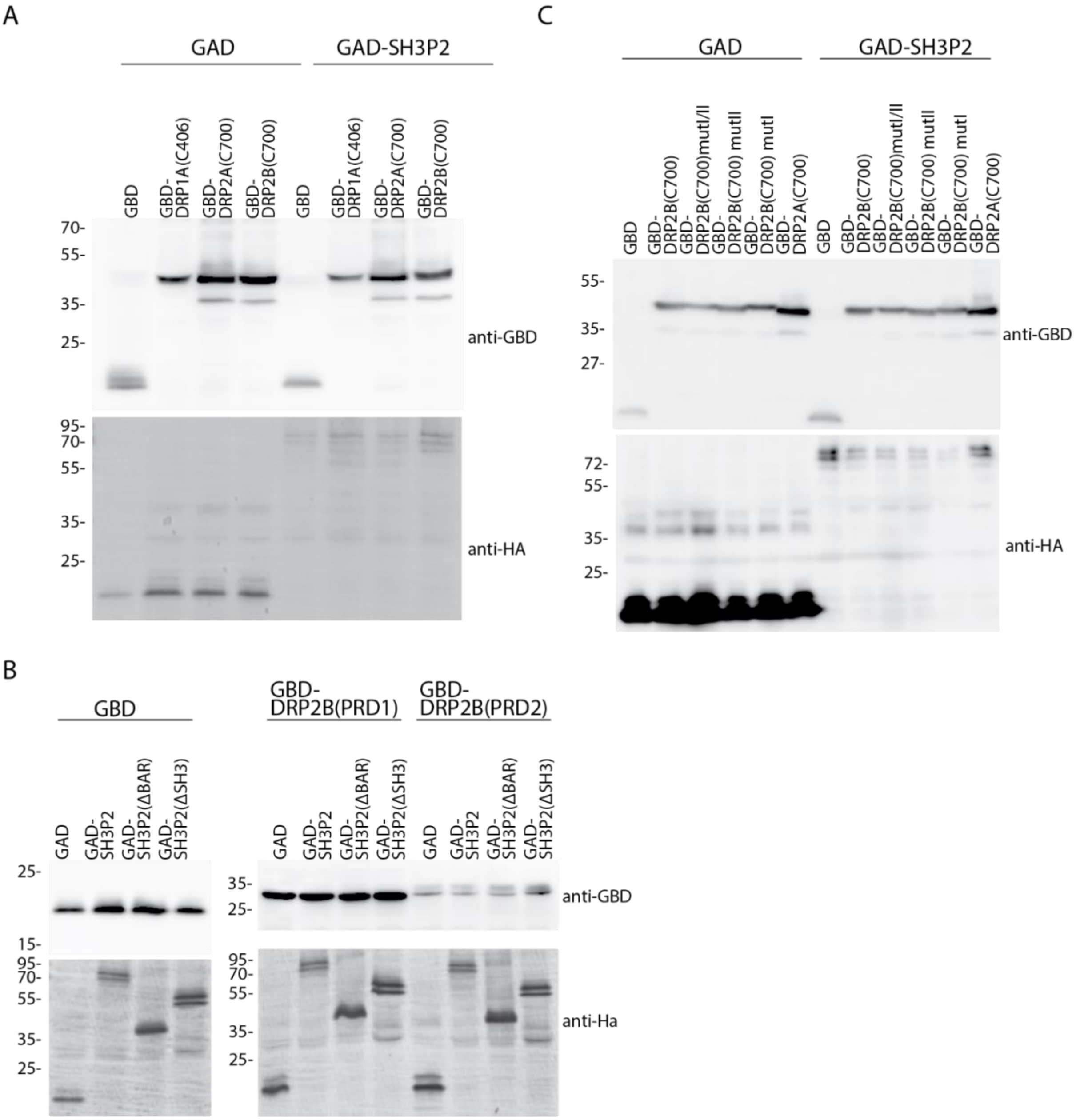

Fig. S4. Expression of YTH constructs. (A, B, C) Total proteins were extracted from yeast cells used for YTH analyses in Fig. 4B and subjected to immuno- blotting using anti-GAL4BD and an anti-HA antibody. The anti-HA antibody was used for the detection of GAD-fusion proteins that also contain an HA-tag.

Journal of Cell Science • Supplementary information

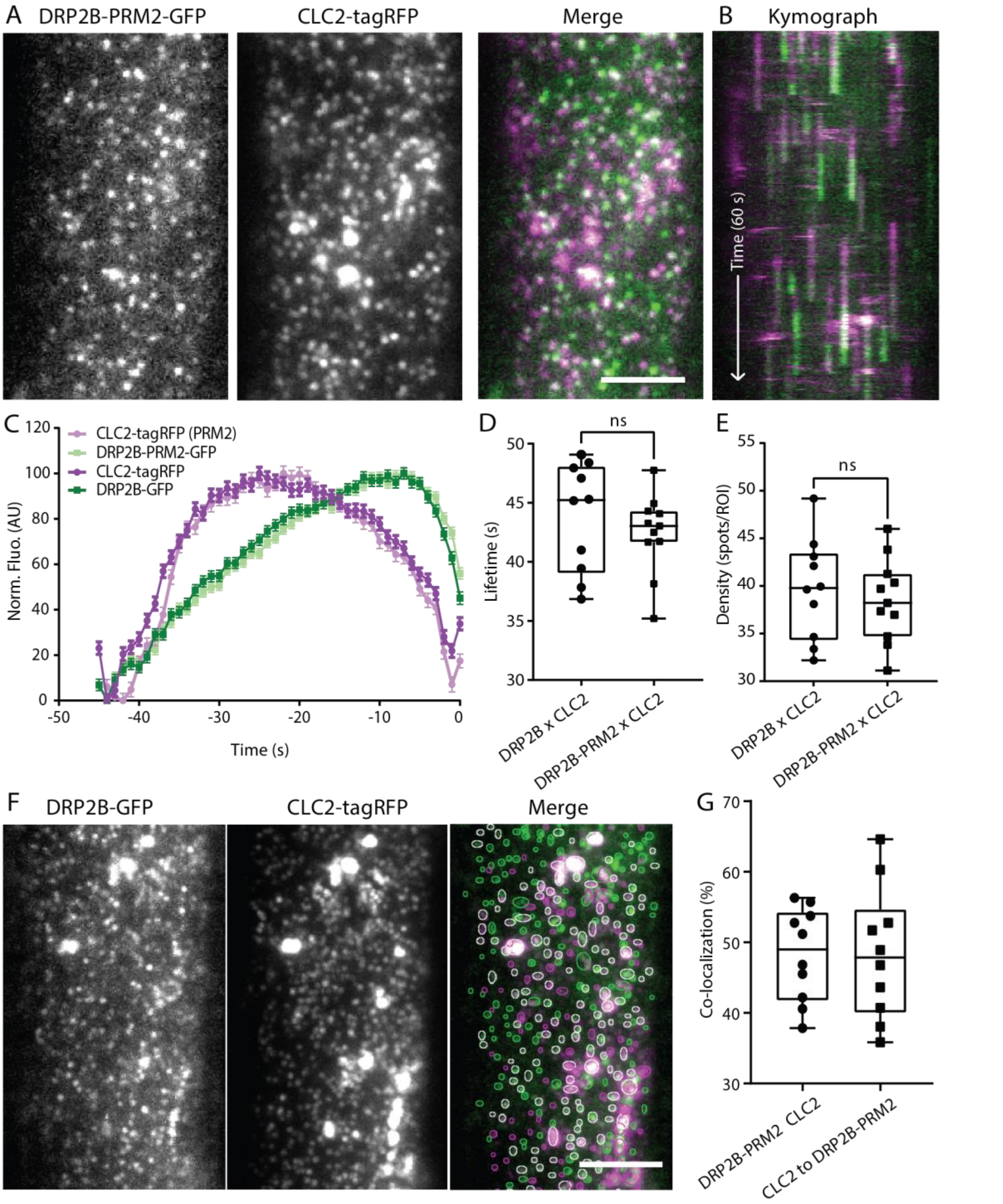

Fig. S5. Dynamics of DRP2B-PRM2 x CLC2 *in vivo*. (A) TIRF-M images of a cell surface of root epidermal cell expressing fluorescently tagged *pDRP2B::DRP2B-PRM2-GFP* and *pRPS5A::CLC2-tagRFP* (*drp2b-2-/-*). Scale bar: 5 µm. (B) Representative kymograph of DRP2B-PRM2 and CLC2 lifetimes on the PM. Arrow represents the time direction and the length of 60 sec. (C-E) Data from eleven independent experiments for DRP2B-PRM2 x CLC2 were combined and ten independent experiments for DRP2B x CLC2 to generate a (C) mean recruitment profile of DRP2B and DRP2B-PRM2 foci, (D) mean lifetime (DRP2B, 43.81±1.45

Journal of Cell Science • Supplementary information

s; DRP2B-PRM2, 42.41±1.01s), and (E) mean density lifetime (DRP2B, 39.67±1.67 spots ROI^{-1}; DRP2B-PRM2, 38.49±1.31 spots ROI^{-1}) of CME events. Plots indicate Mean±SEM, DRP2B x CLC2, n=10 cells from independent roots, 27,647 tracks; DRP2B-PRM2 x CLC2, n=11 cells from independent roots, 36,486 tracks. Plot, Mean ± SEM, ns >0.05, t-test to compare to control. (F) Representative TIRF-M image of co-localisation analysis of DRP2B-PRM2-GFP and CLC2-tagRFP foci. Scale bar: 5 µm. (G) Quantification of co-localised spots. 48.26±2.1% of DRP2B-PRM2 were co-localised to CLC2, and 48.32.5±2.9% of CLC2 were co-localised to DRP2B-PRM2.

Journal of Cell Science • Supplementary information

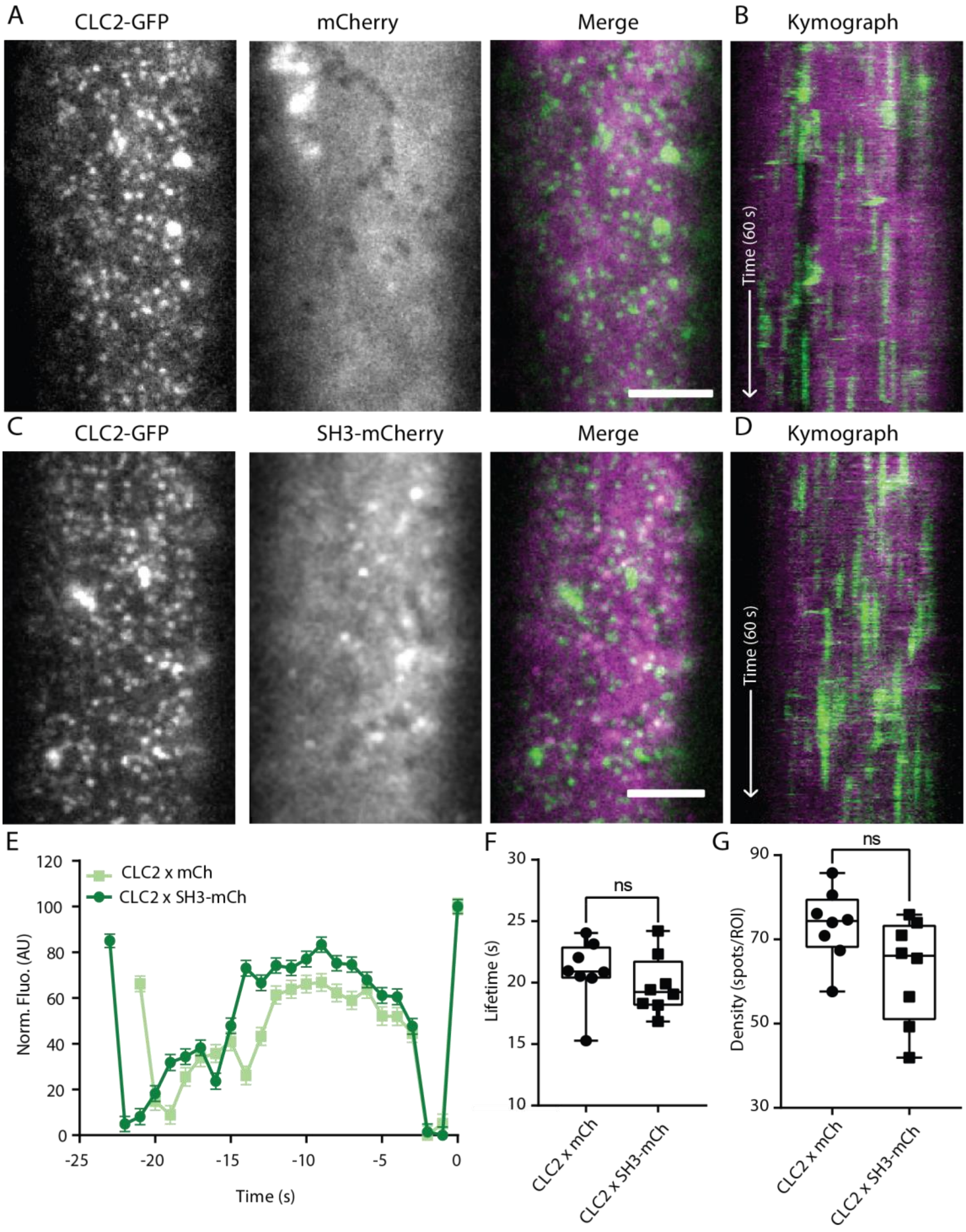

Fig. S6. Overexpression of the SH3 domain does not influence the dynamics of CLC2 *in vivo*. (A) TIRF-M images of a cell surface of root epidermal cell expressing fluorescently tagged *pRPS5A::CLC2-GFP* and free *pXVE:UBQ10::mCherry*. Scale bar: 5 µm. (B) Representative kymograph of CLC2 lifetimes on the PM after 24h mCherry overexpression. Arrow represents the time direction and the length of 60 sec. (C) TIRF-M images of a cell surface of root epidermal cell expressing fluorescently tagged CLC2 and SH3-mCherry of SH3P2. Scale bar: 5 µm. (D) Representative kymograph of CLC2 lifetimes on the PM after

Journal of Cell Science • Supplementary information

24h SH3-mCherry overexpression. Arrow represents the time direction and the length of 60 sec. (E-G) Data from eight independent experiments for CLC2 x mCherry and eight independent experiments for CLC2 x SH3-mCherry were combined to generate a (C) mean recruitment profiles of CLC2 foci, (D) mean lifetime (CLC2 x mCherry, 20.9±0.92 s; CLC2 x SH3-mCherry, 19.78±0.8s), and (E) mean density lifetime (CLC2 x mCherry, 73.38.67±3 spots ROI^{-1}; CLC2 x SH3-mCherry, 62.58±4.3 spots ROI^{-1}) of CME events. Plots indicate Mean±SEM, CLC2 x mCherry, n=8 cells from independent roots, 91,321 tracks; CLC2 x SH3-mCherry, n=8 cells from independent roots, 40,268 tracks. Plot, Mean ± SEM, ns >0.05, t-test to compare to control.

Journal of Cell Science • Supplementary information

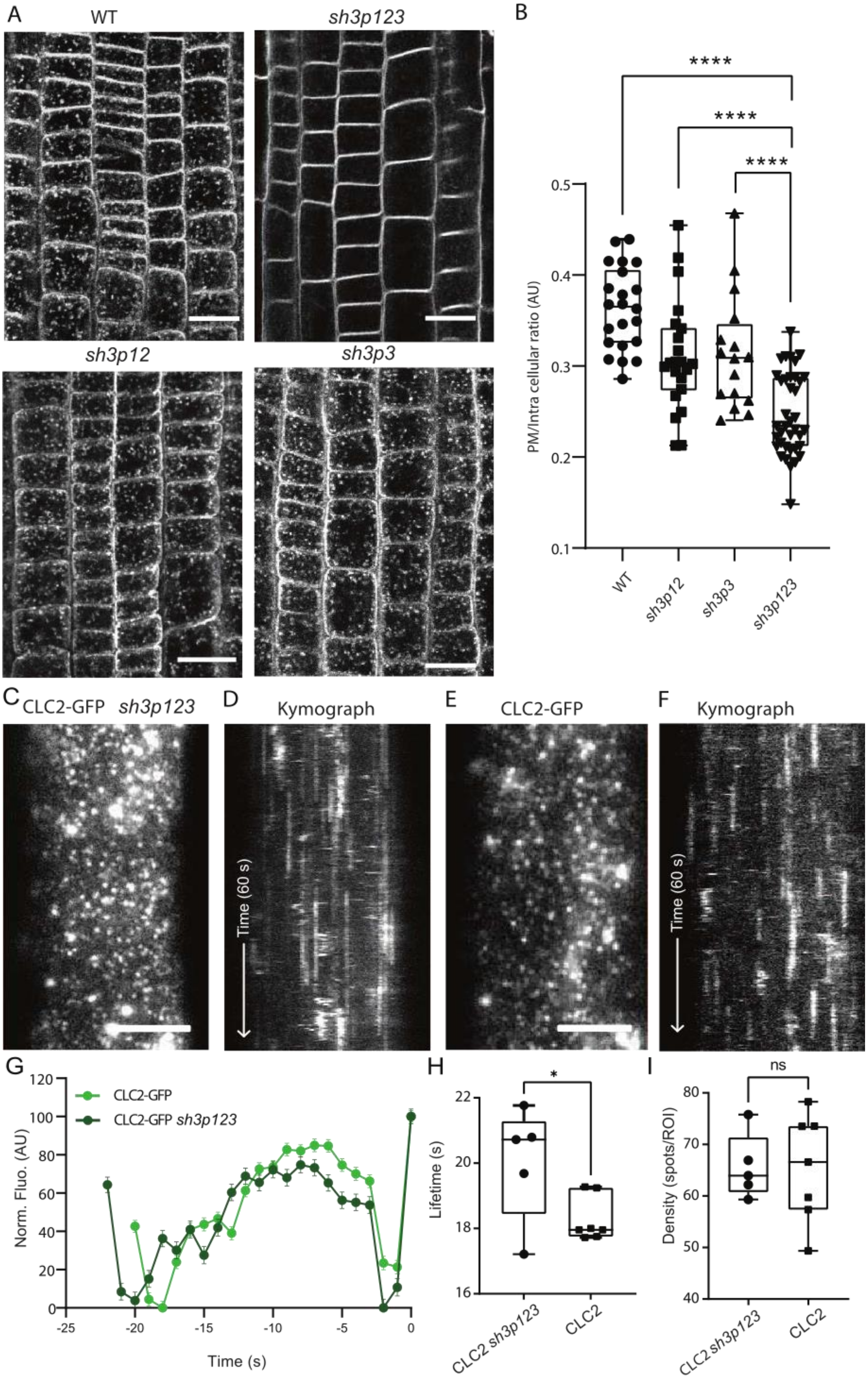

Journal of Cell Science • Supplementary information

Fig. S7. Membrane uptake and CLC2 dynamics in *sh3p123* mutant. (A) Confocal images of epidermal root cells of *A.thaliana* Col-0 (WT), *sh3p12*, *sh3p3*, *sh3p123*, treated with 2 µM membrane dye FM4-64 15 min, RT. Scale bar: 20 µm. (B) Quantification of the membrane uptake. Plots indicate Mean±SEM. For WT, n=22 independent seedlings, 697 cells; for *sh3p12*, n=22 independent seedlings, 582 cells; *sh3p3*, n=16 independent seedlings, 417 cells; *sh3p123*, n=35 independent seedlings, 1060 cells. Plot, Mean ± SEM, ****P < 0.001, t-test to compare to control. (C) TIRF-M image of a cell surface of root epidermal cell expressing *pRPS5A::CLC2-GFP* in *sh3p123* background and (D) a representative kymograph of CLC2 lifetime on the PM. (E) TIRF-M image of a cell surface of root epidermal cell expressing CLC2 and (F) a representative kymograph of CLC2 (Col-0) lifetime on the PM. Scale bar: 5 µm. Arrow represents the time direction and the length of 60 sec. (G-I) Data from seven independent experiments for CLC2-GFP and five independent experiments for CLC2 *sh3p123* were combined to generate a (G) mean recruitment profile of CLC2 and CLC2 *sh3p123* foci, (H) mean lifetime (CLC2, 18.27±0.25 s; CLC2 *sh3p123*, 20.04±0.77 s), and (I) mean density (CLC2, 65.48±3.9 spots ROI^{-1}; CLC2 *sh3p123*, 65.63±2.8 spots ROI^{-1}) of CME events. Plots indicate Mean±SEM CLC2, n=7 cells from independent roots, 46,995 tracks; CLC2 *sh3p123*, n=5 cells from independent roots, 44,205 tracks. Plot, Mean ± SEM, *P < 0.0432, ns>0.05, t-test to compare to control.

Journal of Cell Science • Supplementary information

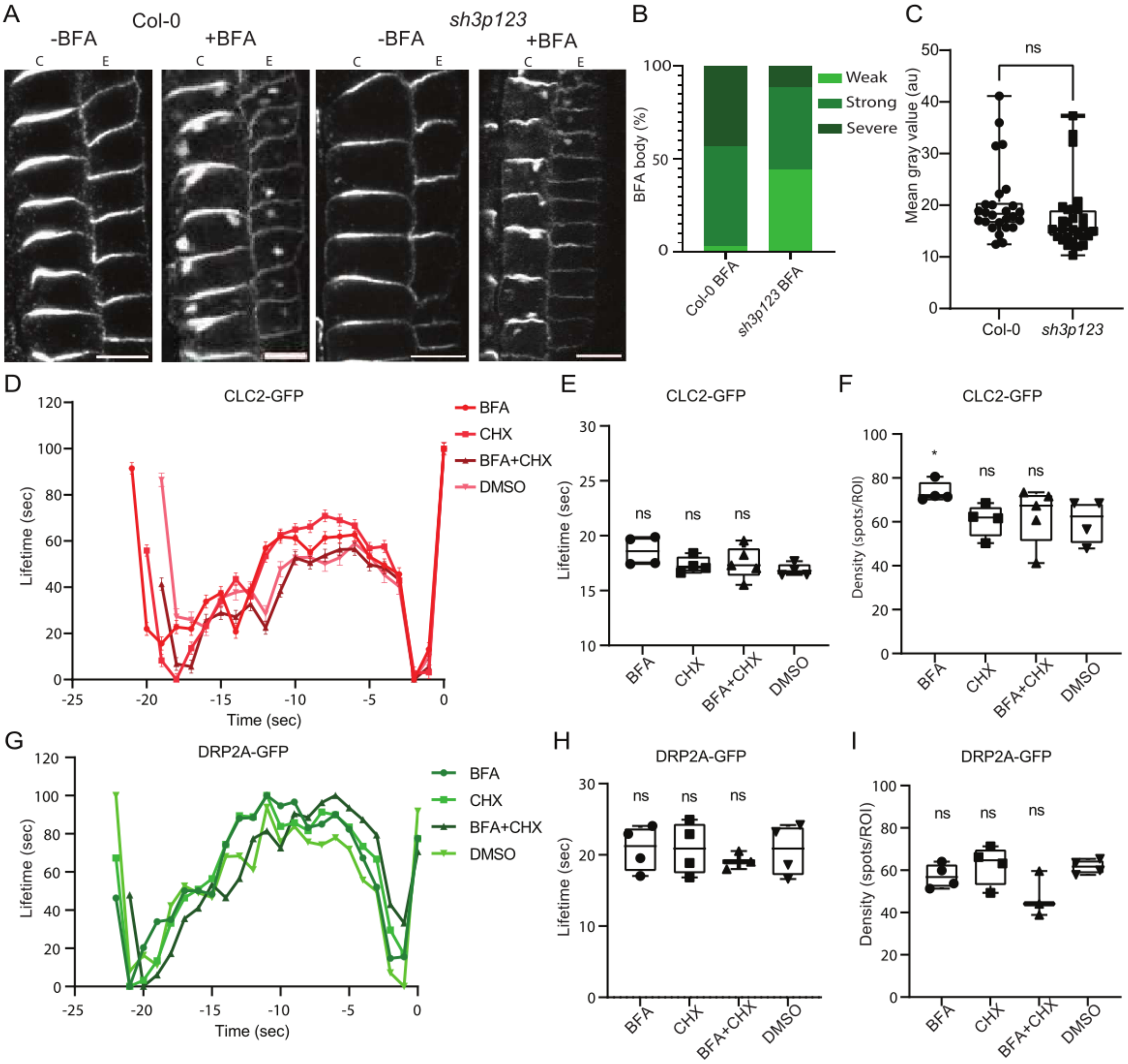

Fig. S8. Influence of BFA and CHX on the endocytosis rates in *Arabidopsis*. (A) Confocal images of PIN2 localization in Epidermis E and Cortex C cells of *Arabidopsis* roots untreated or treated with 50 µM BFA for 1 h in Col-0 or *sh3p123* triple mutant. Scale bar 20 µm. (B) A stacked column chart representing the percentage of roots, per genotype, distributed in three different categories (weak, strong, severe) based on the abundance of PIN2 BFA bodies (internalization) formed in the cells after BFA treatment. (C) The comparison of PIN2 membrane signal in untreated samples. Col-0, n=26 roots, *sh3p123*, n=28 roots, ns>0.05, t-test. (D-F) Results from TIRF images of *pRPS5A::CLC2-GFP* treated with BFA, CHX, combination of BFA and CHX or DMSO. (D) A mean recruitment profile of CLC2, (E) mean lifetime (BFA, 18.75±0.07 s; CHX, 17.24±0.06s, BFA+CHX, 17.65±0.06 s; DMSO, 16.76±0.09s), and (F) mean density (BFA, 73.6±1.9 spots ROI^{-1}; CHX, 60.68±2.3 spots ROI^{-1}, BFA+CHX, 62.89±1.4 spots ROI^{-1}; DMSO, 60.31±2.3 spots ROI^{-1}) of CME events. Plots indicate Mean±SEM BFA, n=4 cells from independent roots, 62,301 tracks; CHX, n=4 cells

Journal of Cell Science • Supplementary information

from independent roots, 48,942 tracks, BFA+CHX, n=5 cells from independent roots, 71,955 tracks; DMSO, n=4 cells from independent roots, 28,288 tracks. Plot, Mean ± SEM. Brown-Forsythe and Welch one-way ANOVA test was performed, with Dunnett's multiple comparison post hoc test to compare each genotype to the wild-type values. *, P value < 0.05, ns – no statistical significance. (G-I) Results from TIRF images of *pDRP2A::DRP2A-GFP* treated with BFA, CHX, combination of BFA and CHX or DMSO. (G) A mean recruitment profile of DRP2A, (H) mean lifetime (BFA, 19.5±0.14 s; CHX, 20.45±0.17s, BFA+CHX, 19.01±0.17 s; DMSO, 19.93±0.15s), and (I) mean density (BFA, 57.21±2.1 spots ROI^{-1}; CHX, 62.49±3.2 spots ROI^{-1}, BFA+CHX, 47.54±0.9 spots ROI^{-1}; DMSO, 61.63±2.7 spots ROI^{-1}) of CME events. Plots indicate Mean±SEM BFA, n=4 cells from independent roots, 19,650 tracks; CHX, n=4 cells from independent roots, 16,332 tracks, BFA+CHX, n=3 cells from independent roots, 13,450 tracks; DMSO, n=4 cells from independent roots, 21,247 tracks. Plot, Mean ± SEM. Brown-Forsythe and Welch one-way ANOVA test was performed, with Dunnett's multiple comparison post hoc test to compare each genotype to DMSO. ns – no statistical significance.

Journal of Cell Science • Supplementary information

Supplem. for fig.4

Marker: page ruler pre stained plus from Thermofisher anti-GBD

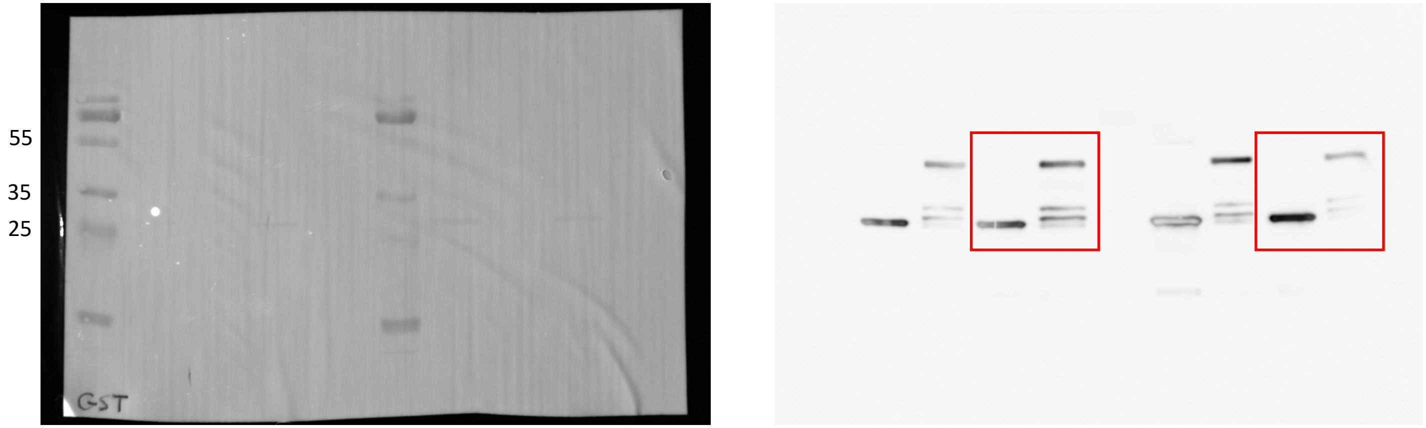

Marker

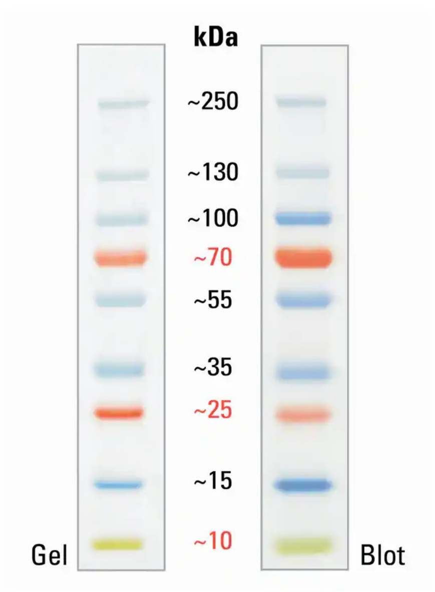

anti-SH3P2

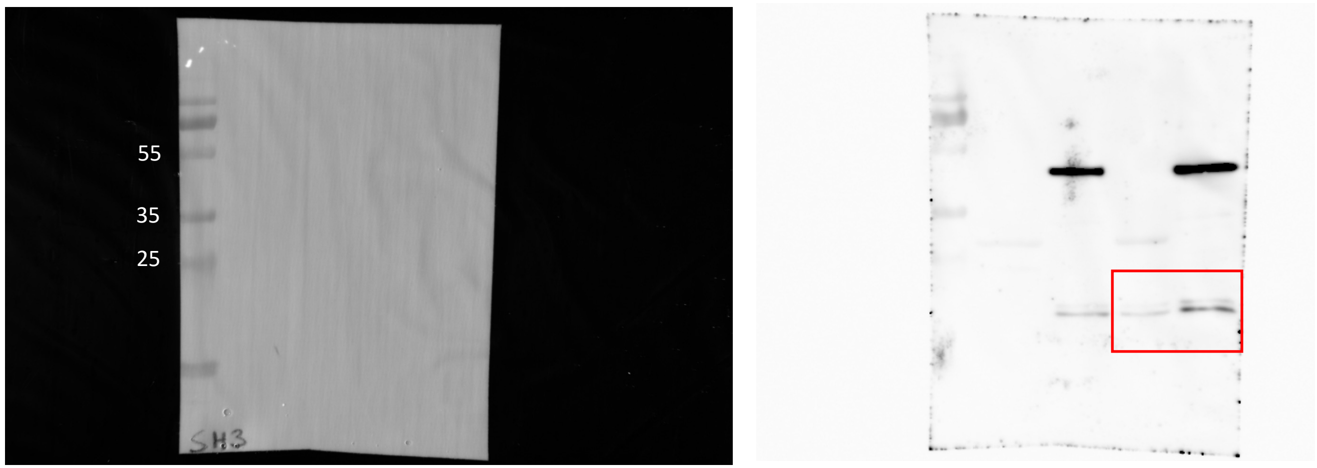

anti-MBP

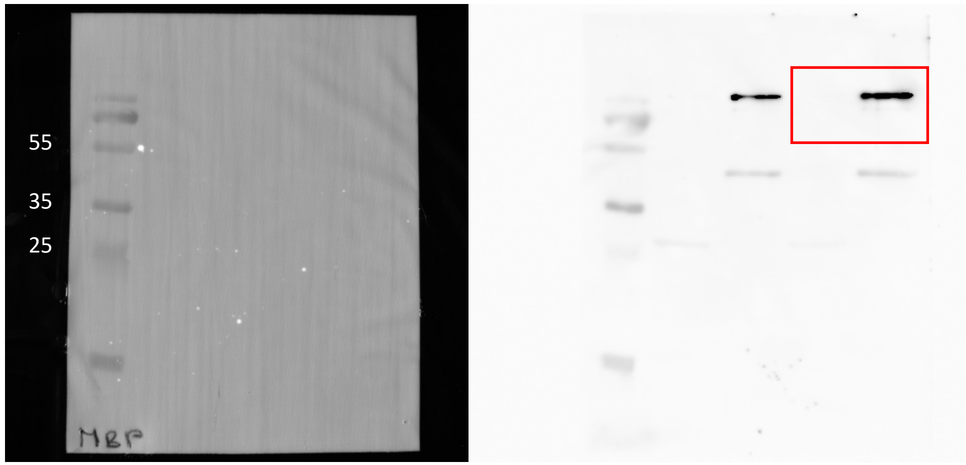

Journal of Cell Science • Supplementary information

Supplem. for fig.S4A

Marker: page ruler pre stained plus from Thermofisher anti-GBD

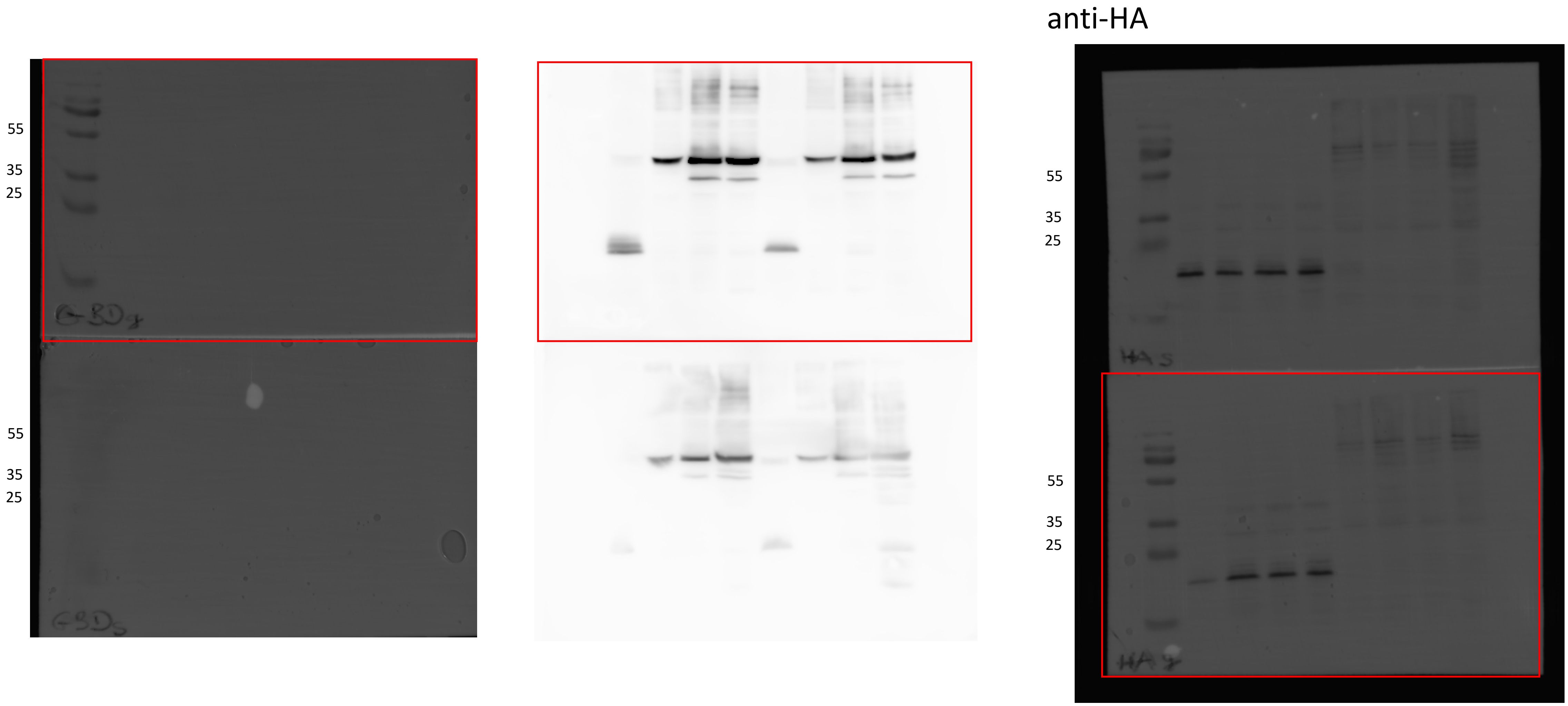

Journal of Cell Science • Supplementary information

J. Cell Sci.: doi:10.1242/jcs.261720: Supplementary information

Supplem. for fig.S4B

Marker: page ruler pre stained plus from Thermofisher anti-GBD

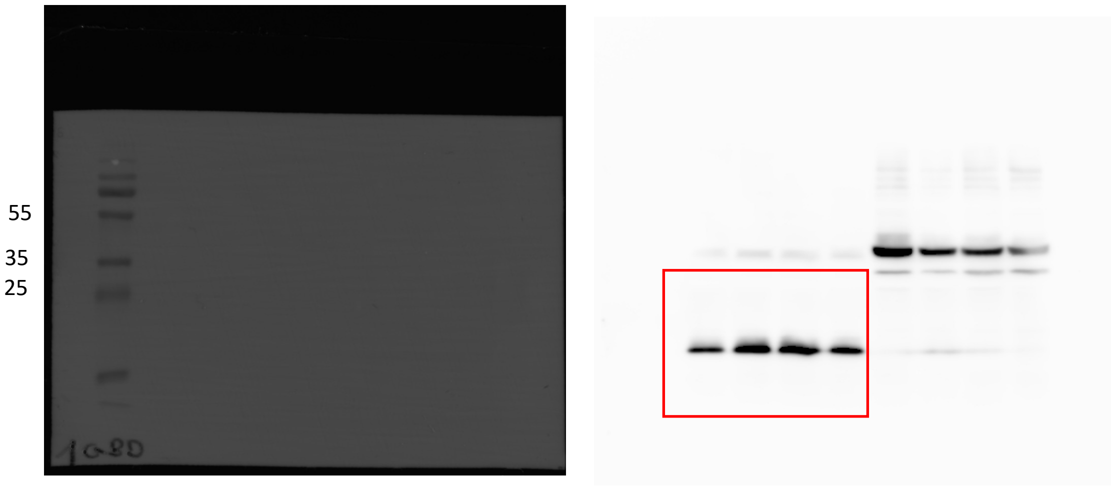

anti-GBD

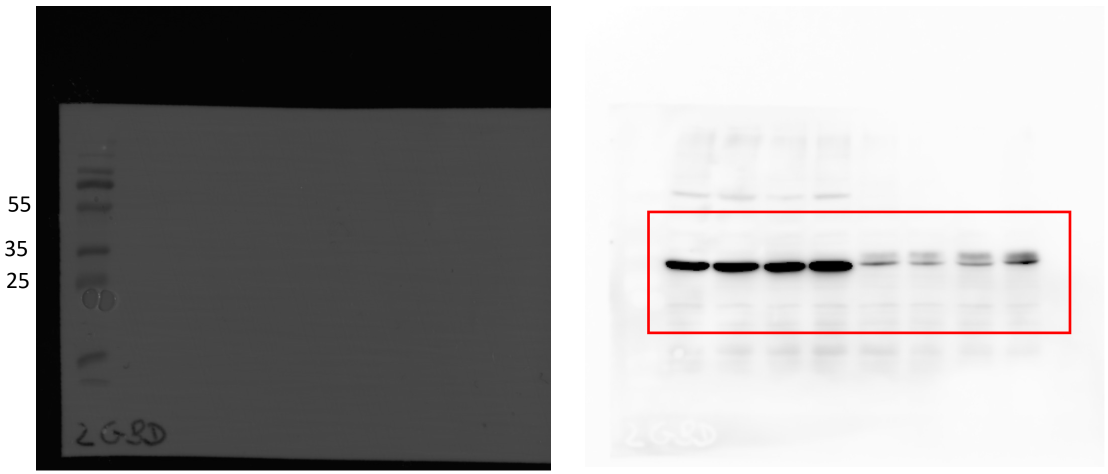

anti-HA

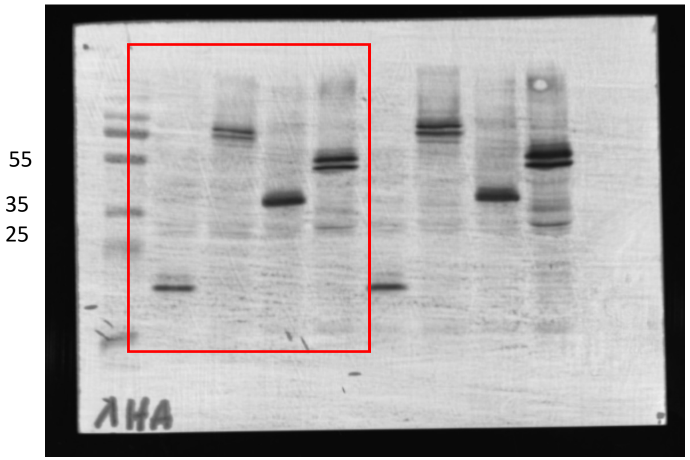

anti-HA

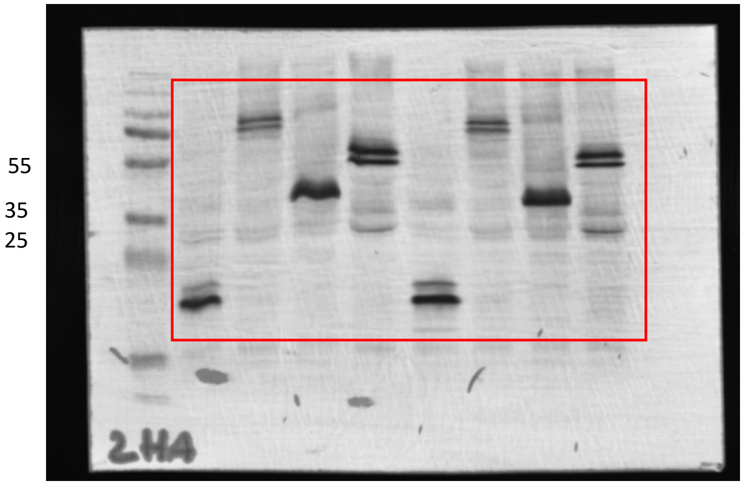

Journal of Cell Science • Supplementary information

Supplem. for fig.S4C

Marker: page ruler pre stained plus from Thermofisher

anti-GBD

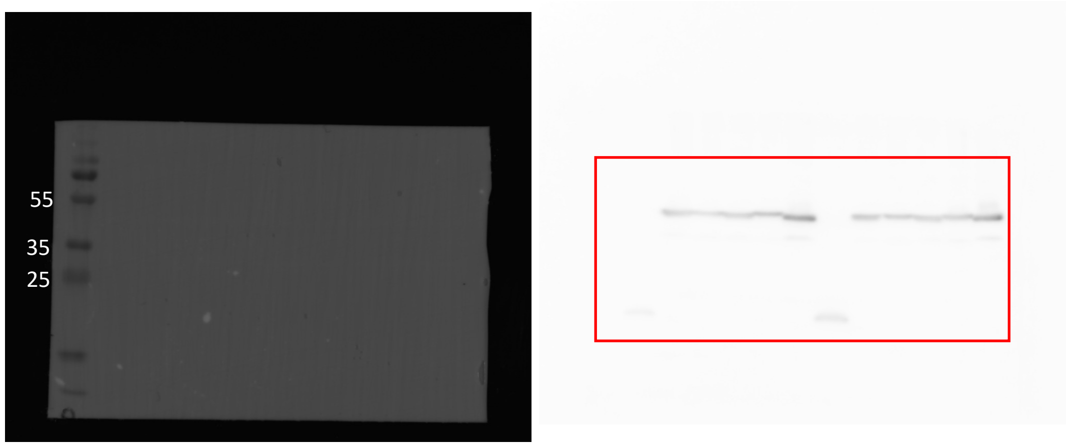

anti-HA

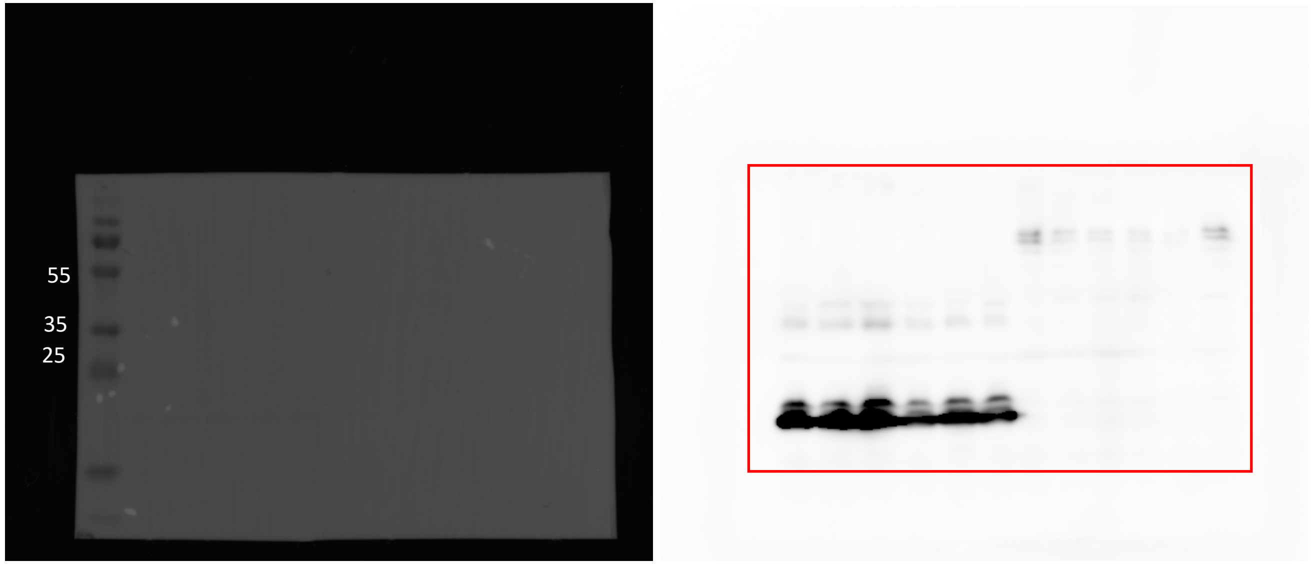

Fig. S9. Blot Transparency

Journal of Cell Science • Supplementary information

Table S1. Codon optimized AtSH3P2 and primers used

Primer name	Primer sequence
pTB146_fwd	TCGAGCCCGGGTGACTGCAGG
pTB146_rev	ACCACCAATCTGTTCTCTGTGAGCCTC
codOpt_SH3P2	ATGGAGGATAACGATATTATTGAGGCTCACAGAGAACAGATTGGTGGTGATGCA ATACGGAAGCAGGCCTCGCGGCTGCGCGAGCAGGTAGCTAGACAGCAACAAGC TGTGTTTAAACAGTTTGGAGGTGGGGGCTACGGCAGCGGCTTGGCAGACGAGGC TGAACTGAACCAACATCAGAAACTTGAGAAGCTGTATATTTCTACACGGGCGGC AAAACACTATCAACGCGACATCGTTCGGGGCGTCGAAGGGTATATTGTTACTGG AAGCAAGCAAGTCGAAATCGGTACAAAGCTGTCAGAAGACAGTCGTAAATACG GTAGTGAAAACACCTGTACAAACGGAAATGTGTTGACCCGTGCAGCGTTGAACT ATGGTCGCGCCAGAGCTCAAATGGAAAAGGAACGGGGTAATATGCTGAAAGCG CTTGGAACGCAAGTGGCAGAACCTCTTCGGGCCATGGTGCTTGGGGCACCGTTG GAAGACGCGCGCCATCTGGCTCAACGCTATGATCGCATGAGACAAGAAGCTGAA GCTCAGGCGACAGAGGTGGCACGTCGGCAGGCAAAGGCTCGTGAATCGCAAGG CAACCCGGACATATTGATGAAGTTAGAATCTGCGGAAGCAAAACTTCACGATCT TAAAAGCAATATGACCATATTAGGAAAGGAGGCAGCATCTGCACTGGCGTCAGT AGAAGACCAACAGCAAAAACTGACGTTAGAACGTCTGTTGTCTATGGTGGAGTC CGAAAGAGCTTACCATCAGAGAGTTTTACAGATACTTGATCAGCTTGAGGGCGA AATGGTTTCAGAGCGTCAGCGCATTGAGGCGCCATCCACACCCAGTTCAGCGGA CTCGATGCCACCTCCTCCATCGTACGAGGAGGCAAACGGAGTTTTCGCCAGTCA AATGCATGACACTTCTACCGACTCTATGGGATACTTTCTGGGGGAGGTATTGTTC CCATACCACGGGGTAACAGATGTCGAATTGAGCTTATCCACGGGTGAGTACGTT GTTGTGCGCAAGGTCACCGGTTCTGGATGGGCTGAAGGGGAGTGTAAAGGGAAG GCGGGATGGTTTCCTTACGGGTACATTGAAAGACGTGAGCGTGTATTGGCTTCA AAAGTTAGCGAGGTATTCTGATCGAGCCCGGGTGACTGCAGGAAGGGGATCCGG CTGCTA
DRPR2A_prom_fw	GGGGACAACTTTGTATAGAAAAGTTGTTGTCCGCAGATCTTGCC
DRPR2A_prom_rev	GGGGACTGCTTTTTTGTACAAACTTGACGCGACTAGCAAAAGC
DRPR2B_prom_fw	GGGGACTGCTTTTTTGTACAAACTTGACTTCTTCCACTGTACACACT
DRPR2B_prom_rev	GGGGACAACTTTTCTATACAAAGTTGTCTGTTTTGCCGTTCAAAGAGA
Drp2aGDNAAttB1	GGGGACAAGTTTGTACAAAAAAGCAGGCTTCATGGAGGCGATCGATGAGTT
Drp2aGDNAAttB2	GGGGACCACTTTGTACAAGAAAGCTGGGTAATACCTATAAGCTGAACCTG
Drp2bGDNAAttB1	GGGGACAAGTTTGTACAAAAAAGCAGGCTTCATGGAGGCGATCGATGAGTT
Drp2bGDNAAttB2	GGGGACCACTTTGTACAAGAAAGCTGGGTACTAATACCTGTAAGATGATC
Drp2a p902 903a F	GCGGTGGTGCTGCTGCCAACCGATTGGG
Drp2a p902 903a R	CCCAATCGGTTGGCAGCAGCACCACCGC
Drp2b p908 909a F	GTGGCGGAGCTGCTGCGAGCCGGTTTGG
Drp2b p908 909a F	CCAAACCGGCTCGCAGCAGCTCCGCCAC
SH3_dom_SH3P2_fwd	GGGGACAAGTTTGTACAAAAAAGCAGGCTTTATGGCATCTCAGATGCATGACA
SH3_dom_SH3P2_rev	GGGGACCACTTTGTACAAGAAAGCTGGGTAGAAAACTTCGGACACTTTGC
MN176 SH3P2(C257-369)fw EcoRI	AAGGGAATTCATGTCTGAGAGGCAACGTATAG
MN177 SH3P2(N1-257)rv BamHI	AAGGGGATCCTCATACCATCTCTCCTTCGAG

Journal of Cell Science • Supplementary information

MN226 AT5G42080 XhoI rv	AAGGCTCGAGTCACTTGGACCAAGCA
MN255 DRP2A rv XhoI	AAGGCTCGAGCTAATACCTATAAGCTGAACCTGTAG
MN256 DRP2B(aa747) fw EcoRI	AAGGGAATTCTATGTTGAAGCTGTTCTCAAC
MN315 DRP2B (EcoRI) rv	AAGGGAATTCCTAATACCTGTAAGATGATCCAG
MN316 DRP2A(C700) fw (NdeI)	AAGGCATATGATGGGCCAAGTGGGCAGTG
MN317 DRP2B(C700) fw (NdeI)	AAGGCATATGATGAAGGTTATCCAGGCCC
MN321 DRP2A rv (EcoRI)	AAGGGAATTCCTAATACCTATAAGCTGAACCTGTAG
MN349 DRP1A (NdeI) fw	AAGGCATATGGGTTACCGTCGTCTCATTGA
MN350 DRP2B (700-800) rv (EcoRI)	AAGGGAATTCCTAGTTCTGATCCTCTTGAATCAATG
MN351 DRP2B(C837) fw (NdeI)	AAGGCATATGATGGACAATAGTGGCACTGAAAG
MS11 SH3P2 fw EcoRI	GGAAGAATTCAGTGATGCAATTAGAAAACA
MS12 SH3P2 rv SalI	GGAAGTCGACTCAGAAAACTTCGGACACT

Journal of Cell Science • Supplementary information
