# Supplementary material for: Role of the dynamin-related protein 2 family and SH3P2 in clathrin-mediated endocytosis in Arabidopsis thaliana
Source: J Cell Sci. 2024 May 2;137(8):jcs261720. doi: 10.1242/jcs.261720 (PMC11112126; doi:10.1242/jcs.261720)
Supplement: Supplementary file 1 [file joces-137-261720_review_history.pdf]

Role of Dynamin-Related Proteins 2 and SH3P2 in Clathrin-Mediated Endocytosis in *Arabidopsis thaliana*

Nataliia Gnyliukh, Alexander Johnson, Marie-Kristin Nagel, Aline Monzer, David Babic, Annamaria Hlavata, Saqer S. Alotaibi, Erika Isono, Martin Loose and Jiri Friml
DOI: 10.1242/jcs.261720

Editor: David Stephens

Review timeline
Original submission: 11 October 2023
Editorial decision: 24 November 2023
First revision received: 30 January 2024
Accepted: 12 March 2024

Original submission

First decision letter

MS ID#: JOCES/2023/261720

MS TITLE: Role of Dynamin-Related Proteins 2 and SH3P2 in Clathrin-Mediated Endocytosis in Plants

AUTHORS: Nataliia Gnyliukh, Alexander Johnson, Marie-Kristin Nagel, Aline Monzer, Annamaria Hlavata, Erika Isono, Martin Loose, and Jiri Friml

ARTICLE TYPE: Research Article

We have now reached a decision on the above manuscript.

To see the reviewers' reports and a copy of this decision letter, please go to: https://submit-jcs.biologists.org and click on the 'Manuscripts with Decisions' queue in the Author Area. (Corresponding author only has access to reviews.)

As you will see, the reviewers raise a number of substantial criticisms that prevent me from accepting the paper at this stage. They suggest, however, that a revised version might prove acceptable, if you can address their concerns. I consider their comments to be fair and detailed and that they suggest revisions that would clarify and strengthen the conclusions. These include the framing of the comparison between species.

If you think that you can deal satisfactorily with the criticisms on revision, I would be pleased to see a revised manuscript. We would then return it to the reviewers.

Please ensure that you clearly highlight all changes made in the revised manuscript. Please avoid using 'Tracked changes' in Word files as these are lost in PDF conversion.

I should be grateful if you would also provide a point-by-point response detailing how you have dealt with the points raised by the reviewers in the 'Response to Reviewers' box. Please attend to all of the reviewers' comments. If you do not agree with any of their criticisms or suggestions please explain clearly why this is so.

© 2024. Published by The Company of Biologists under the terms of the Creative Commons Attribution License (https://creativecommons.org/licenses/by/4.0/).

Reviewer 1

Advance summary and potential significance to field

Gnyliukh and colleagues investigate the role of DRP2 and SH3P2 in clathrin-mediated endocytosis (CME) in Arabidopsis by using high resolution in vivo imaging. They present some compelling evidence that DRP2 recruitment to the site of endocytosis is independent of the SH3P2. Although containing some negative results, this study is of interest as it reports differences between plant and mammalian CME models and it raises further questions about the function of SH3P2 proteins in plants.

Comments for the author

I have several suggestions for improvement before publication:
> The tile should be limited to Arabidopsis
> In the introduction pg. 3 also cite the work from Van Damme lab on TPC function as an adaptor not only as membrane bending factor
> Show the execution of the DRP2A-GFP complementation by including information on ovule abortion phenotype of the complemented lines.
> Note in the legends the respective promoters and backgrounds of the transgenic lines. For example, the complemented drp2 mutants were not used for the vivo imaging thus, expression levels can interfere with the CME dynamics.
> Fig. S3 duplicates Fig. S2 and the data described in the manuscript (S3A-D)
pg. 8 are missing therefore difficult to judge, the authors must include the data
> The title of Fig. 4 is related to DRP2A but in the cartoon in panel A DRP2B is shown, perhaps is best to show DRP2A or both.
> In Fig. 4A please include only information relevant for the YTH result sown in Fig. 4B. It is not clear what exactly is C700, C406 and C747 (the last in Fig.
5). Moreover was the SH3P2 used in the YTH experiment (Fig. 4B) a full length or a truncated version as shown in Fig. 4A. For clarity, my suggestion is to combine Fig. 4A, B and Fig. 5A, B.
> Were the PRMs of DRP2A tested in YTH? The authors presumed that DRP2B and DRP2A are interchangeable but their PRM sequences are different. Was the interaction between SH3P2 and PRM mutants tested by GST pull downs?
> Are the SH3P2-GFP/tagRFP fusions functional? Was the dynamics of SH3P2 and DRP2B analyzed?
> The tile of Fig. 5 states that PRM2 is important for interaction with SH3P2 in vitro and in vivo, whereas the first claim is supported by the YTH experiments (only for DRP2B), the second is based on evaluation the dynamics between the PRM2 mutants and CLC2. In my opinion, the second conclusion here is that the mutations in the second PRMs do not interfere with DRP2 dynamics in vivo. The in vivo interaction was not tested for example by Co-IP or other methods.
> The sh3p123 mutant has been reported in the preprint doi:
https://doi.org/10.1101/2022.01.07.475403 Col-0 9C but I could not find the description and the molecular characterization of this mutant. It is advisable that such data are shown to the reviewers. Moreover, the phenotype differs from that of the published RNAi line as also noted by the authors.

Reviewer 2

Advance summary and potential significance to field

The authors have explored the role of two proteins namely Dynamin Related Protein 2 (DRP2) and SH3 domain containing Protein 2 (SH3P2), in Clathrin Mediated endocytosis (CME), in epidermal Root cells of Arabidopsis Thaliana. DRP2A and B are homologous to mammalian Dynamin and are observed to co-localise with Clathrin light chain in plants. The SH3P2 proteins have a BAR domain that recognises curvature and the Src homology-3 (SH3) domain which can bind proline rich domains (PRD) in proteins such as DRP2.

The authors observed a significant co-localisation (60%) of DRP2A-GFP and Clathrin light chain 2 (CLC2)-RFP foci and determine the recruitment profiles of both these markers. They observe that DRP2A intensity increased just before disappearance of CLC2 foci indicating recruitment before

© 2024. Published by The Company of Biologists under the terms of the Creative Commons Attribution License (https://creativecommons.org/licenses/by/4.0/).

scission. They further investigated the co-localisation and determined recruitment profiles of SH3P2-GFP and CLC2-mOrange protein at individual CME events. About 60% of CLC2 foci were also positive for SH3P2 and its recruitment profile was similar to DRP2A. Hence the authors suggest that both DRP2A and SH3P2 have a role in CME vesicle scission in plants, similar to dynamin and BAR-domain proteins (Endophillin and Amphiphysin) in CME in animal and fungi.

By TEM microscopy they observed tubulation in DOPC+DOPS+PIP (4,5) P2 LUVs when purified SH3P2 is added, as compared to no protein control. Hence the authors propose that SH3P2 can aid in membrane deformation at CME sites which occur preferentially in region on plasma membrane enriched with negatively charged lipids.

DRP2A has two Proline Rich Motifs (PRM) for potential interaction with SH3P2.
Using YTH assays, point mutations and pull down assays, they observed that PRM2 at the C terminus of DRP2A and B is necessary for interaction with SH3P2. Co-localisation and lifetime analysis of DR2PA GFP and SH3P2-RFP puncta in TIRF revealed that about 34% of DRP2A puncta were positive for SH3P2 and around 40% of SH3P2 puncta were positive for DRP2A indicating that a larger fraction of puncta of each of these proteins did not colocalise. The lifetime of the foci positive for both proteins was about half the lifetime of a CLC2 puncta indicating their potential recruitment before scission.

However, point mutation in the PRM2 of DRP2A did not affect the membrane recruitment and lifetime of DRP2A. It only slightly reduced the lifetime of CLC2 foci and had no effect on the number of CLC2 foci. CLC2 RFP colocalised to the same extent with the mutant protein when compared to wild type DRP2A. The authors hypothesised that expression of SH3 domain only in the cytosol would sequester DRP2A and affect CLC2 dynamics, but overexpression of this protein did not affect CLC2 dynamics.

The next set of experiments are carried out in a triple knockout like of SH3P 1, 2 and 3 to rule out compensatory effects by other isoforms. The recruitment and lifetime of DRP2A was not affected even in the triple negative SH3P line. Moreover even the lifetime of CLC2 RFP and of the CME adaptor protein TPL (which would mark only successful CME events) was also not affected. Endocytosis was measured by labelling cell membrane with FM4-64 and puncta per cell were counted. There was a reduction in FM4-64 puncta in the triple negative SH3P line indicating an overall decrease in membrane uptake. SH3P2 can bind ubiquitinated proteins such as PIN2 which also undergoe uptake and recycling through CME, hence this was tested for but found to be unaffected in the SH3P2 triple negative line.

The data is rationalized by suggesting that despite the structural homology and binding characteristics of CLC, DRP2 and SH3P2, they function in a very different manner from their animal and fungal cell counterparts, leaving open the possibility of new ways to generate the endocytic vesicle using similar but not identical components. Although the authors do not provide any good alternatives the discrepancies with the well characterized counterparts of these proteins indicate that further research is required to understand even a well characterized and purportedly conserved mechanism such as CME.

Comments for the author

Before this manuscript's conclusions may be fully justified, there is a key concern and a number of specific and more pervasive lacunae that will need to be rectified.

Key concern:
While there is experimental documentation of an endocytic defect in DRP2A/ DRP2B knock out (ie in the drp2a-/-; drp2b-/-) background. An experiment is required to join the dots between plants cells requiring CLC2 as well as DRP2A/B in executing CME. If the drp2a-/-; drp2b-/- mutant cells exhibit a serous deficit in PIN2 endocytosis which correlates with an expected dynamin defect and this is accompanied by alterations in the lifetime of the CLC2 punctae, the remainder of the manuscript will provide a strong argument for the existence of divergent mechanisms of endocytosis despite the conservation of a core machinery- ie clathrin (CLC2) and dynamin (DRP2A/B).

© 2024. Published by The Company of Biologists under the terms of the Creative Commons Attribution License (https://creativecommons.org/licenses/by/4.0/).

Other Comments:

1. Missing data:
The authors have explored the role of SH3P2 in DRP2B recruitment at cell surface during CME by using TIRF microscopy in plants cells. In-vitro reconstitution assays were carried out to determine if SH3P2 can bind and deform membranes.
However, the data on purification and characterisation of SH3P2 protein by mass spectrometry is missing from the supplementary. It is referred to as Fig S3 but the figure panel S2 is re-pasted as Fig S3. Yeast two hybrid (YTH) assays and pull down assays were done to asses physical binding between SH3P2 and DRP2 proteins.
But this entire data set is missing from the supplementary.

The authors mention that through sedimentation assay of Large Unilamelar Vesicles (LUVs) they observe that SH3P2 binds vesicles with negatively charged lipids such as PA or PIP (4,5) P2 as compared to control DOPC only or DOPC+DOPS vesicles.
This data set is also supposed to be a part of Fig S3 and is missing.

2. Supplementary FigS7: The recruitment and lifetime of DRP2A and DR2PB must be checked after SH3 domain expression in cytosol. This can shed some light on the possibility of compensatory mechanisms by either of the isoform of DRP2 to cause the normal recruitment of CLC2 observed in S7

3. Fig7 PIN2 internalization:
i) It is possible that Brefeldin A (BFA) and cyclohexamide (CHX) treatments may reduce secretion and synthesis, respectively of other crucial proteins required for CME. Therefore, the lack of any effect on PIN2 endocytosis and recycling may be a consequence of generally impaired CLC2 and DRP2A recruitment and expression on the membrane post CHX and BFA; therefor the triple mutants may have no additional phenotypes. This could be checked and may serve as a control for PIN2 uptake.

ii) How do the surface levels and ubiquitination of PIN2 in the triple knockout of SH3P compare to wild type? This needs to be assessed and taken into consideration while quantifying internalisation or recycling.

iii) The quantification of PIN2 recycling/uptake is categorised based on severity of phenotype (7B). What is the phenotype being scored for and how are the categories of weak, strong or severe decided quantitatively and how is this data set correlated to the endocytic defect described in S7?

iv) In the case of PIN2, it is possible genetic perturbations reveal the possibility of PIN2 utilizing other pathways. Co-localisation of the PIN2 puncta with the CME adaptor TPL which may provide some confirmation of this possibility.
The endocytic effect can be tested/confirmed upon ligand stimulation or other CME plant cargo like cellulose synthase.

4) Additional:
i) In the text the authors refer to Fig 1D, E to refer to data for TPL and CLC2 (top page 7) endocytosis events, but this data is not available in Figure 1D, E (or anywhere else in this manuscript).

ii) Relevant to all figures documenting puncta-lifetime and density per ROI data:
The authors should spell out the interpretation of the kymograph? These details need to be mentioned in the results or legend. Do the images represent a single cell or a part or an array of epidermal root cells is not clear. What exactly is the size of the ROI in density plots and how many such ROIs were imaged per cell/experiment is not clear. The authors indicate the number of tracks used in some experiments but not all As the quantification of foci per ROI is used for calculating density of endocytic events, these details have to be mentioned in legends or methods.

The fact that there are many puncta that reappear in the same kymograph, indicates that there could be hot-spots for endocytosis. This aspect should be characterized here.

© 2024. Published by The Company of Biologists under the terms of the Creative Commons Attribution License (https://creativecommons.org/licenses/by/4.0/).

iii) In general the writing is a little sloppy and the connection of the experiments with the legends in the Figures somewhat difficult to parse.

5) Overall comments:

A more than frivolous criticism of this manuscript is that the authors first set up a strawman and then show that this is indeed a non-tenable argument. They expect that considering some superficial similarities between the endocytic machineries of the plant and animal kingdom - proteins which have been named due to varying degrees of similarity/homology must have the same function. Thus, it is not surprising that at some level of the construction of the endocytic process the systems begin to diverge. Considering that there are a numerous modules that are part of the endocytic process, perhaps plant cells have reconfigured these processes and therefore their pathways may not conform to what is observed in more well studied systems such as animal cells. A similar argument has been made in the study of the differences between endocytosis in animal cells and yeast. While the processes appear superficially similar, and share several common features, the pathways may differ in their molecular detail. This alone is not particularly interesting, unless these results provide an understanding of the mechanism of endocytosis in the plant system under study.

Since SH3P2 physically interacts with DRP2A and triple knockdown causes a reduction in FM4-64 uptake, it may play a potential role in a Clathrin-independent endocytic process via a different endocytic pathway that does not utilize either Clathrin or dynamin. While the authors claim that the role of Endophilin or SH3P2 in CIE in plants remain as yet undiscovered, several papers have provided compelling evidence for this pathway in root hair tip cells.

First revision

Author response to reviewers' comments

Response to the reviewers

Dear prof. David Stephens,
We thank for the opportunity to submit our revised draft of the manuscript "Role of Dynamin-Related Proteins 2 and SH3P2 in Clathrin-Mediated Endocytosis in Plants" for the publication in *Journal of Cell Science*. We highly appreciate the time and effort that you and the reviewers put into providing the feedback on our manuscript. We are thankful for the valuable comments and improvements to our manuscript. We have incorporated most of the suggested corrections, which are highlighted within the manuscript. All page numbers refer to the revised manuscript file and changes are highlighted in yellow.

Reviewer 1 Advance Summary and Potential Significance to Field:
Gnyliukh and colleagues investigate the role of DRP2 and SH3P2 in clathrin- mediated endocytosis (CME) in Arabidopsis by using high resolution in vivo imaging. They present some compelling evidence that DRP2 recruitment to the site of endocytosis is independent of the SH3P2. Although containing some negative results, this study is of interest as it reports differences between plant and mammalian CME models and it raises further questions about the function of SH3P2 proteins in plants.

Reviewer 1 Comments for the Author:
I have several suggestions for improvement before publication:
> The tile should be limited to Arabidopsis

Author response: Thank you for the suggestion. We changed the title to "Role of Dynamin-Related Proteins 2 and SH3P2 in Clathrin-Mediated Endocytosis in *Arabidopsis thaliana*" as suggested.
> In the introduction pg. 3 also cite the work from Van Damme lab on TPC function as an adaptor

© 2024. Published by The Company of Biologists under the terms of the Creative Commons Attribution License (https://creativecommons.org/licenses/by/4.0/).

not only as membrane bending factor
Author response: Thank you for the suggestion. The reference was added to the introduction.

> Show the execution of the DRP2A-GFP complementation by including information on ovule abortion phenotype of the complemented lines.

Author response: As suggested by the reviewer, we added the characterization of ovule abortion phenotype in *drp2a-1-/-;drp2b-2+/-* mutant and mutant complemented line with pDRP2A::DRP2A-GFP in the Supplem. Fig. S1A-B.

> Note in the legends the respective promoters and backgrounds of the transgenic lines. For example, the complemented drp2 mutants were not used for the vivo imaging thus, expression levels can interfere with the CME dynamics.

Author response: Thank you for this suggestion. We clarified genetic background for all plant lines that were used in the Methods section (Plant material and growth conditions) and added the information for the Fig. 1, 2, 5, 6 and Supplem. Fig. S1, S2, S5, S6, S7. All changes are marked with a yellow highlighter.

> Fig. S3 duplicates Fig. S2 and the data described in the manuscript (S3A-D) pg. 8 are missing therefore difficult to judge, the authors must include the data

Author response: Thank you for the comment. We apologize for this mistake. The correct data is in the Fig. S3.

> The title of Fig. 4 is related to DRP2A but in the cartoon in panel A DRP2B is shown, perhaps is best to show DRP2A or both.

Author response: We thank the reviewer for the suggestion. This was corrected in the Fig. 4.

> In Fig. 4A please include only information relevant for the YTH result sown in Fig. 4B. It is not clear what exactly is C700, C406 and C747 (the last in Fig.5). Moreover was the SH3P2 used in the YTH experiment (Fig. 4B) a full length or a truncated version as shown in Fig. 4A. For clarity, my suggestion is to combine Fig. 4A, B and Fig. 5A, B.

Author response: Following the reviewers' suggestion, we changed the arrangement in the figures, splitting the *in vitro* interaction assay (Fig. 4), DRP2AxSH3P2 in vivo dynamics (Fig. 5), and *in vivo* imaging of DRP2A-PRM2 mutant (Fig. 6). Main text was re-arranged accordingly.

> Were the PRMs of DRP2A tested in YTH? The authors presumed that DRP2B and DRP2A are interchangeable but their PRM sequences are different. Was the interaction between SH3P2 and PRM mutants tested by GST pull downs?

Author response: Thank you for the comment. No, we have the Y2H data of minimal domain analyses just for DRP2B. Since the core motif for interaction is conserved: consensus (R/K)xxPxxP or consensus PxxPx(R/K) (Ball et al., 2005, Recognition of Proline-Rich Motifs by Protein-Protein-Interaction Domains, Angewandte Chemie; BJ Mayer, 2001, SH3 domains: complexity in moderation, J Cell Sci); we assumed the difference in amino acids should not influence the interaction between SH3 domain and PRMs of DRP2A and DRP2B (the interaction should be comparable).

Regarding the GST pull down - the PRD mutant variants could not be purified from *E.coli* despite a lot of optimization efforts. Therefore, we decided to do the mutant and minimal domain analysis with Y2H.

> Are the SH3P2-GFP/tagRFP fusions functional? Was the dynamics of SH3P2 and DRP2B analyzed?

Author response: The complementation of SH3P2-GFP construct is shown in Nagel et al. 2017, PNAS.

© 2024. Published by The Company of Biologists under the terms of the Creative Commons Attribution License (https://creativecommons.org/licenses/by/4.0/).

SH3P2-tagRFP is exactly the same construct just GFP is exchanged with RFP, therefore they should behave similar. We were aiming to analyze the dynamics of DRP2B together with SH3P2, similarly as done for DRP2Ax SH3P2 in Fig. 5; however, unfortunately, we failed to obtain plant line that was expressing both fluorescently tagged proteins sufficiently strong for performing TIRF microscopy. Therefore, we were limited to DRP2A, but judging from the complementation experiment, very high level of homology between DRP2A and DRP2B, similar dynamics, and the functional redundancy, we do not expect drastic difference between DRP2A and DRP2B.

> The tile of Fig. 5 states that PRM2 is important for interaction with SH3P2 in vitro and in vivo, whereas the first claim is supported by the YTH experiments (only for DRP2B), the second is based on evaluation the dynamics between the PRM2 mutants and CLC2. In my opinion, the second conclusion here is that the mutations in the second PRMs do not interfere with DRP2 dynamics in vivo. The in vivo interaction was not tested for example by Co-IP or other methods.

Author response: Thank you for the suggestion. We corrected the conclusions according to the new panel arrangement and the reviewer suggestion.

> The sh3p123 mutant has been reported in the preprint doi: https://doi.org/10.1101/2022.01.07.475403 Col-0 9C but I could not find the description and the molecular characterization of this mutant. It is advisable that such data are shown to the reviewers. Moreover, the phenotype differs from that of the published RNAi line as also noted by the authors.

Author response: Thank you for pointing this out. Since, this information is a part of an intellectual property that belongs to another author and it will be eventually included in the updated preprint, we are now including it as information for the reviewers.

sh3p1,2,3

SH3P1
ttgggggtagataggattgttgaggtatgaattggttatagttaggtgaaaagtatgggactttttcttgttttagacatagattttgccttttttgtt
ttgattttaggtttagattaccttctaataatatcagacaagattgtgttatttgtgtaggtttctttatttgtattaaaaaaataatctggtttttgtg
cagGCGGTTTTGAAGCATTTAGGGCATGTTAATGCTGATGCTGTTGTTGTTGATGAAGAAGAGCTTCATTGTCAT
CAGAAACTTCAAGATCTTTATAGCTCTACTAAAGCTGCTAAGgtaaagatattttaggtttttttgttgaggctttctcggataata
atctctttagttttggcttatcaaagttgttctgttcatagCGTTTGCAAAGGAACATTGTTCGTGGACTCGAAGGTTTCATTGC
GACTGGCACAAAGGTTGTTGAGATAGgtatgttcttgtttcctacttcatctcatatagtgcttcttggaatcacatttgtaatggtgtat
gtccatagGGTTGAAGTTTGCTGAAGATTTCAAGAAATATGGAGATGAGAATCCTGATGCTAATACTCCTCTATCAA
GA GTCGCACATCACTTTGGGACCTCTTACAAGTCGGTGGAAGGCGAGAGGGAAACTCT

sh3p1
ttgggggtagataggattgttgaggtatgaattggttatagttaggtgaaaagtatgggactttttcttgttttagacatagattttgccttttttgtt
ttgattttaggtttagattaccttctaataatatcagacaagattgtgttatttgtgtaggtttctttatttgtattaaaaaaataatctggtttttgtg
cagGCGGTTTTGAAGCATTTAGGGCATGTTAATGCTGATGCTGTTGTTGTTGATGAAGAAGAGCTTCATTGTCAT
CAGAAACTTCAAGATCTTTATAGCTCTACTAAAGCTGCTAAGgtaaagatattttaggtttttttgttgaggctttctcggataata
atctctttagttttggcttatcaaagttgttctgttcatagCGTTTGGCAAAGGAACATTGTTCGTGGACTCGAAGGTTTCATTG
CGACTGGCACAAAGGTTGTTGAGATAGgtatgttcttgtttcctacttcatctcatatagtgcttcttggaatcacatttgtaatggtgt
atgtccatagGGTTGAAGTTTGCTGAAGATTTCAAGAAATATGGAGATGAGAATCCTGATGCTAATACTCCTCTATC
AAG AGTCGCACATCACTTTGGGACCTCTTACAAGTCGGTGGAAGGCGAGAGGGAAACTCT

SH3P1-cF ttgggggtagataggattgttg
SH3P1-cR AGAGTTTCCCTCTCGCCTTC

CAPS genotyping:
SH3P1-cF + cR
HpyCH4V
WT 408+264
sh3p1 673

SH3P2
Tcgtgttcgaaagcagagaaatttgttctgagctcaacgccatttttgacgatttcgaatctctgtcttcctgagttgttccaagtaa

© 2024. Published by The Company of Biologists under the terms of the Creative Commons Attribution License (https://creativecommons.org/licenses/by/4.0/).

tcggagggaactcgtaaagtggaagaataggagaaagagagaaggaagaagatttgtgaatagctcttgatctggtgaagaATGGATGCAA
TTAGAAAACAAGCTAGCAGGTTGAGAGAACAAGTTGCGAGACAGCAGCAGgttaaagttcttaacttttggtttgcccatttg
cttagatcccgattctgtatctttttttctagattttgtttttatcttctcttgtgattccgttgagtcgttggtttctggtgaatgaatcaataatgtga
tgatgataaagggg ctagctttttttcggatcgatggtttttgacttaatgagtaattggatctgagtgtgattaatacgttgattcgatg
tgagattgggtaaaattctgtagaattggagaagtttttgtcaaaggg

sh3p2

tcgtgttcgaaagcagagaaatttgttctgagctcaacgccatttttgacgatttcgaatctctgtcttctctgagttgttccaagtaa
tcggagggaactcgtaaagtggaagaataggagaaagagagaaggaagaagatttgtgaatagctcttgatctggtgaagaATGGATGCAA
TTAGAAAACAAGCTAGCAGGTTGAGAGAACAAGTTGCGAGACAGCAAGCAGgttaaagttcttaacttttggtttgcccattt
gcttagatcccgattctgtatctttttttctagattttgtttttatcttctcttgtgattccgttgagtcgttggtttctggtgaatgaatcaataatgtg
atgatgataaaggg gctagctttttttcggatcgatggtttttgacttaatgagtaattggatctgagtgtgattaatacgttgattcgat
gtgagattgggtaaaattctgtagaattggagaagtttttgtcaaaggg

SH3P2-cF tcgtgttcgaaagcagagaa
SH3P2-cR ccctttgacaaaaacttctcca

CAPS genotyping:
SH3P2-cF + cR
Fnu4HI, TseI, ApeKI
WT 226 +
277
sh3p2 504

SH3P3 (sh3p3 SALK_065790*)*
ttgttggggggagaaggtttcagtatgattcatcgtagcacaataccctcaaacatatgttattgtcactttcagGAGTTCCAGAGAGATATT
GTTAAAGCAGCTGAGGCATTTACCACGATCGGGCTCAGACACATTGAAGCAGgtacttaactacttatgagtgtctgttttg
tgttacctatcattgaattaagttatactatgatacttttgtgagttttcttttaatattctttaaaatttcagGAACCAAGTTGTCTGAAGAC
TGTTGTAGGTATGGGAACGAAAATAGTCAGAACATTGATGAGAATATACTGGCCAAGGCTGCAGCTATATACGG
TGATGCACGGAAACATGTAGACAAAGAACAAGAAGACTTCAACAAGCTTTTGGCTTCTCAGgttcggcttggattatctg
tgtttaatcttagtatgtttacgtagaattaaatctagtagataacaagagaaaacctttttgttaaatgccgttaagGTTTTGGATCCTCTA
AGAGCTATGGTTGCCGGTTCTCCTCTTGAAGATGCTCGTCATCTTGCTCAACGTTACAGCCGCATGAGACAAGAA
GCAGAGACACATgtaatcatcttaagatcttagttctatgatgatgttggctattttattttgtaatgtaagttaaaacttaaaagcaaaacag
GCAACGGAAGTCTCTAGACGGCAAGCACGTGTAAGAGAAGCTCCGATTCCAGAAAACGTAGCAAAGCTTCAA
CTAGCGGAAGCAAAAATGCAAGAGCTAAAAGCAAATATGGCAGTCCTTGGTAAAGAAGCAACTGCTGCATTAGCC
GCTGTTGAATCACAGCAACATAGACTCACTTTCCAGAGGCTTGTGGCTATGgtatgttttacatacacacacactggatttttt
ttgtcatatgcagattactatttaccttatcgcttattctagGTTGAAGGGGAGAAGAATTATCACTTAAGAATAGCAGCCATCC
TCAGTGACATTGAAGCCGAGgttagtattggatgaatctttcccacatctttttctcatcaaaggctcacacttttggtcttaccgcgtagA
TGGTAACCGAGAAACAACACAAAGAATCTGCTCCTCCTGCGATTCCAACAGAGAACGGCTCAGAGAAAACGTCAT
ACTTTCTTGCTGAAgtaagttttatcttccgtaa

sh3p3-F GGCTCAGACACATTGAAGCA
sh3p3-R acgcggtaagaccaaaagtg

Reviewer 2 Advance Summary and Potential Significance to Field:

The authors have explored the role of two proteins namely Dynamin Related Protein 2 (DRP2) and SH3 domain containing Protein 2 (SH3P2), in Clathrin Mediated endocytosis (CME), in epidermal Root cells of Arabidopsis Thaliana. DRP2A and B are homologous to mammalian Dynamin and are observed to co- localise with Clathrin light chain in plants. The SH3P2 proteins have a BAR domain that recognises curvature and the Src homology-3 (SH3) domain which can bind proline rich domains (PRD) in proteins such as DRP2.

The authors observed a significant co-localisation (60%) of DRP2A-GFP and Clathrin light chain 2 (CLC2)-RFP foci and determine the recruitment profiles of both these markers. They observe that DRP2A intensity increased just before disappearance of CLC2 foci indicating recruitment before scission. They further investigated the co-localisation and determined recruitment profiles of SH3P2-GFP and CLC2- mOrange protein at individual CME events. About 60% of CLC2 foci were also positive for SH3P2 and its recruitment profile was similar to DRP2A. Hence the authors suggest that both DRP2A and SH3P2 have a role in CME vesicle scission in plants, similar to dynamin and BAR-

© 2024. Published by The Company of Biologists under the terms of the Creative Commons Attribution License (https://creativecommons.org/licenses/by/4.0/).

domain proteins (Endophillin and Amphiphysin) in CME in animal and fungi.

By TEM microscopy they observed tubulation in DOPC+DOPS+PIP (4,5) P2 LUVs when purified SH3P2 is added, as compared to no protein control. Hence the authors propose that SH3P2 can aid in membrane deformation at CME sites which occur preferentially in region on plasma membrane enriched with negatively charged lipids.

DRP2A has two Proline Rich Motifs (PRM) for potential interaction with SH3P2. Using YTH assays, point mutations and pull down assays, they observed that PRM2 at the C terminus of DRP2A and B is necessary for interaction with SH3P2. Co-localisation and lifetime analysis of DR2PA GFP and SH3P2-RFP puncta in TIRF revealed that about 34% of DRP2A puncta were positive for SH3P2 and around 40% of SH3P2 puncta were positive for DRP2A indicating that a larger fraction of puncta of each of these proteins did not colocalise. The lifetime of the foci positive for both proteins was about half the lifetime of a CLC2 puncta indicating their potential recruitment before scission.

However, point mutation in the PRM2 of DRP2A did not affect the membrane recruitment and lifetime of DRP2A. It only slightly reduced the lifetime of CLC2 foci and had no effect on the number of CLC2 foci. CLC2 RFP colocalised to the same extent with the mutant protein when compared to wild type DRP2A. The authors hypothesised that expression of SH3 domain only in the cytosol would sequester DRP2A and affect CLC2 dynamics, but overexpression of this protein did not affect CLC2 dynamics.

The next set of experiments are carried out in a triple knockout like of SH3P 1, 2 and 3 to rule out compensatory effects by other isoforms. The recruitment and lifetime of DRP2A was not affected even in the triple negative SH3P line. Moreover even the lifetime of CLC2 RFP and of the CME adaptor protein TPL (which would mark only successful CME events) was also not affected. Endocytosis was measured by labelling cell membrane with FM4-64 and puncta per cell were counted. There was a reduction in FM4- 64 puncta in the triple negative SH3P line indicating an overall decrease in membrane uptake. SH3P2 can bind ubiquitinated proteins such as PIN2 which also undergoe uptake and recycling through CME, hence this was tested for but found to be unaffected in the SH3P2 triple negative line.

The data is rationalized by suggesting that despite the structural homology and binding characteristics of CLC, DRP2 and SH3P2, they function in a very different manner from their animal and fungal cell counterparts, leaving open the possibility of new ways to generate the endocytic vesicle using similar but not identical components. Although the authors do not provide any good alternatives, the discrepancies with the well characterized counterparts of these proteins indicate that further research is required to understand even a well characterized and purportedly conserved mechanism such as CME.

Reviewer 2 Comments for the Author:

Before this manuscript's conclusions may be fully justified, there is a key concern and a number of specific and more pervasive lacunae that will need to be rectified.

Key concern:

While there is experimental documentation of an endocytic defect in DRP2A/ DRP2B knock out (ie in the drp2a-/-; drp2b-/-) background. An experiment is required to join the dots between plants cells requiring CLC2 as well as DRP2A/B in executing CME. If the drp2a-/-; drp2b-/- mutant cells exhibit a serous deficit in PIN2 endocytosis which correlates with an expected dynamin defect and this is accompanied by alterations in the lifetime of the CLC2 punctae, the remainder of the manuscript will provide a strong argument for the existence of divergent mechanisms of endocytosis despite the conservation of a core machinery- ie clathrin (CLC2) and dynamin (DRP2A/B).

Author response: While we appreciate the reviewer's feedback and we fully agree that it would be very nice to have those data, we cannot perform the suggested experiment. The suggested experiment to check dynamics of CLC2 and/or PIN2 endocytosis in *drp2a-/-; drp2b-/-* double mutant can not be performed due to the gametophyte-lethal phenotype of *drp2a-/-; drp2b-/-*

© 2024. Published by The Company of Biologists under the terms of the Creative Commons Attribution License (https://creativecommons.org/licenses/by/4.0/).

double mutation (Backues et al., 2010). In the heterozygous lines the presence of one of the DRP2 protein has been shown to fully complement the observed phenotypes, due to the functional redundancy of these proteins.

Other Comments:
1. Missing data:
> The authors have explored the role of SH3P2 in DRP2B recruitment at cell surface during CME by using TIRF microscopy in plants cells. In-vitro reconstitution assays were carried out to determine if SH3P2 can bind and deform membranes. However, the data on purification and characterisation of SH3P2 protein by mass spectrometry is missing from the supplementary. It is referred to as Fig S3 but the figure panel S2 is re-pasted as Fig S3.

Author response: Thank you for the comment. We apologize for this mistake. The correct data is in the Fig. S3.

> Yeast two hybrid (YTH) assays and pull down assays were done to asses physical binding between SH3P2 and DRP2 proteins. But this entire data set is missing from the supplementary.

Author response: This set of data is provided in the Fig. 4 as well as supplementary Fig. S4 with expression of YTH constructs.

> The authors mention that through sedimentation assay of Large Unilamelar Vesicles (LUVs) they observe that SH3P2 binds vesicles with negatively charged lipids such as PA or PIP (4,5) P2 as compared to control DOPC only or DOPC+DOPS vesicles. This data set is also supposed to be a part of Fig S3 and is missing.

Author response: Thank you for the comment. We apologize for this mistake. The correct data is in the Fig. S3.

2. Supplementary FigS7: The recruitment and lifetime of DRP2A and DR2PB must be checked after SH3 domain expression in cytosol. This can shed some light on the possibility of compensatory mechanisms by either of the isoform of DRP2 to cause the normal recruitment of CLC2 observed in S7

Author response: We thank the reviewer for this suggestion. However, given the results from the normal CLC2 dynamics under overexpression of SH3 domain (Fig. S6) and normal dynamics of CLC2 and DRP2s with point mutation in PRM2 that disrupts the direct interaction between DRP2s and SH3P2 (Fig. 6, S5), it is unlikely that his experiment would yield additional insights.

3. Fig7 PIN2 internalization:

i) It is possible that Brefeldin A (BFA) and cyclohexamide (CHX) treatments may reduce secretion and synthesis, respectively of other crucial proteins required for CME. Therefore, the lack of any effect on PIN2 endocytosis and recycling may be a consequence of generally impaired CLC2 and DRP2A recruitment and expression on the membrane post CHX and BFA; therefor the triple mutants may have no additional phenotypes. This could be checked and may serve as a control for PIN2 uptake.

Author response: Thank you for the suggested experiment. We tested the influence of the BFA and CHX treatment on the rates of CME using TIRF-M CLC2-GFP and DRP2A-GFP lines. The result of the experiment is presented in the Fig. S8D-I. We detected dynamics and density of the foci similar to what was observed for the DMSO control. Therefore, neither BFA nor CHX have direct influence on the dynamics of CME markers.

ii) How do the surface levels and ubiquitination of PIN2 in the triple knockout of SH3P compare to wild type? This needs to be assessed and taken into consideration while quantifying internalisation or recycling.

Author response: Thank you for the suggested experiment. We did not detect any significant

© 2024. Published by The Company of Biologists under the terms of the Creative Commons Attribution License (https://creativecommons.org/licenses/by/4.0/).

difference when comparing immuno-stained PIN2 in Col-0 and *sh3p123* triple mutant. The data presented in the Fig. S8C.

iii) The quantification of PIN2 recycling/uptake is categorised based on severity of phenotype (7B). What is the phenotype being scored for and how are the categories of weak, strong or severe decided quantitatively and how is this data set correlated to the endocytic defect described in S7?

Author response: Thank you for the question. The phenotype is scored based on the number of PIN2- containing BFA bodies formed in root cells. The categories classify PIN2-containing BFA bodies as being weakly, strongly or severely formed. "Severe" category is given when all the imaged cells of a given root have at least one BFA body, whereas "strong" corresponds when most of the cells have BFA bodies and "weak" scores to the root that have very little or no BFA bodies. The explanation of the evaluation was added to the materials and methods.

iv) In the case of PIN2, it is possible genetic perturbations reveal the possibility of PIN2 utilizing other pathways. Co-localisation of the PIN2 puncta with the CME adaptor TPL which may provide some confirmation of this possibility. The endocytic effect can be tested/confirmed upon ligand stimulation or other CME plant cargo like cellulose synthase.

Author response: Thank you for the suggested experiment. However, it has serious technical limitations that prevent it to be performed. Since PIN2 is a highly-polarized protein and the majority of the protein is located at the basal side of the cells, it can not be visualized using TIRF microscope. Additionally, previous experiments with various ways of interfering with CME show that PINs including PIN2 are internalized to a large extent by CME - CHC HUB overexpression and chc1,2 mutants (Kitakura et al., 2011, Dhonukshe et al., 2007).

4. Additional:

i) In the text the authors refer to Fig 1D, E to refer to data for TPL and CLC2 (top page 7) endocytosis events, but this data is not available in Figure 1D, E (or anywhere else in this manuscript).

Author response: The reference to the lifetime of TPLATE and CLC2 events during CME has been previously studied and therefore is referred to the publication reference at the end of the sentence in Narasimhan et al., 2020. To avoid further confusion, we modified the following sentence as such:

"Average lifetime of these events was 46.3±0.22 s with a density of 39.64±1.68 spots per region of interest [640 μm^2] $(ROI)^{-1}$ (Fig. 1D-E), compared to a long population of endocytosis events represented by TPLATE (TPL) and CLC2 (~43 s) (Narasimhan et al., 2020)."

ii) Relevant to all figures documenting puncta-lifetime and density per ROI data: The authors should spell out the interpretation of the kymograph? These details need to be mentioned in the results or legend. Do the images represent a single cell or a part or an array of epidermal root cells is not clear.

Author response: We thank for the suggestion. We added the kymograph explanation to the results section.

"Representative kymograph visualizes a projection of a random X plane over the time-span of the time laps image."

What exactly is the size of the ROI in density plots and how many such ROIs were imaged per cell/experiment is not clear. The authors indicate the number of tracks used in some experiments but not all As the quantification of foci per ROI is used for calculating density of endocytic events, these details have to be mentioned in legends or methods. The fact that there are many puncta that reappear in the same kymograph, indicates that there could be hot-spots for endocytosis. This aspect should be characterized here.

Author response: Thank you for the question. The exact algorithm of image analysis is described in Johnson et al. 2020. "The density is generated by imposing a 100×100 pixel ROI in the center of

© 2024. Published by The Company of Biologists under the terms of the Creative Commons Attribution License (https://creativecommons.org/licenses/by/4.0/).

the movie, and the mean number of valid tracks is calculated over 100 frames (where the time range of frames used is 50 before the middle time-lapse image to 50 after the middle time-lapse image)" (Johnson et al. 2020). Our imaging data can only let us speculate on the topic of the existence of hot-spots for vesicles emerging; however, no definitive conclusions can be drawn.

iii) In general the writing is a little sloppy and the connection of the experiments with the legends in the Figures somewhat difficult to parse.

Author response: we made it clearer now.

5. Overall comments:
A more than frivolous criticism of this manuscript is that the authors first set up a strawman and then show that this is indeed a non-tenable argument. They expect that considering some superficial similarities between the endocytic machineries of the plant and animal kingdom - proteins which have been named due to varying degrees of similarity/homology must have the same function. Thus, it is not surprising that at some level of the construction of the endocytic process, the systems begin to diverge. Considering that there are a numerous modules that are part of the endocytic process, perhaps plant cells have reconfigured these processes and therefore their pathways may not conform to what is observed in more well studied systems such as animal cells. A similar argument has been made in the study of the differences between endocytosis in animal cells and yeast. While the processes appear superficially similar, and share several common features, the pathways may differ in their molecular detail. This alone is not particularly interesting, unless these results provide an understanding of the mechanism of endocytosis in the plant system under study.

Since SH3P2 physically interacts with DRP2A and triple knockdown causes a reduction in FM4-64 uptake, it may play a potential role in a Clathrin-independent endocytic process via a different endocytic pathway that does not utilize either Clathrin or dynamin. While the authors claim that the role of Endophilin or SH3P2 in CIE in plants remain as yet undiscovered, several papers have provided compelling evidence for this pathway in root hair tip cells.

Author response: We thank the reviewer for their opinion and we in principle agree with the statements, while giving them a slightly different weight.

Firstly, for many decades, studies of the function of proteins involved in different cellular processes have been heavily based on the consideration of the related homologues across different systems. The whole field was "building strawmen"; often with a great success. It cannot dismissed that similar domain organization and protein structure often indicates the similar function. Of course, this is not enough to draw conclusions about the true function of a protein of interest and therefore extensive studies are needed to be performed. This is the exact reason why we performed our research – to experimentally test involvement and function of DRP2s and SH3P2 in plant CME, which was previously suggested by a number of researchers in plant field. We do not think that it is something wrong on the approach to formulate a hypothesis based on protein homology and rigorously test it. We, honestly, do not know how else we could present this data. Without mentioning the SH3P homology to animal counterparts and just present the functional analysis? We believe that this is only matter of taste.

Secondly, regarding the statement about "superficial" similarities between mammalian and yeast CME – CME is an extremely evolutionary conserved vital process, in which currently more than 60 protein homologues have been described to have identical/similar function during vesicle formation. Obviously, the physiological differences of these systems result in differences in some of the aspects of CME mechanism. However, these similarities provide initial tools and ideas for the researcher to study the inevitable differences. This is why the study of the mechanism of plant CME is particularly important, since, as the reviewer mentioned and we fully agree, we cannot simply assume the function of the proteins based on knowledge from other systems.

Lastly, regarding CIE in plants. We are not sure, whether we understand reviewer's comment on the involvement of Endophilin in plant CIE, since this protein is a mammalian homologue of plant SH3P proteins and is not present in plants. As the main research focus of our study is plant CME, we merely discussed the possibility of studied proteins been involved in CIE as we got some

© 2024. Published by The Company of Biologists under the terms of the Creative Commons Attribution License (https://creativecommons.org/licenses/by/4.0/).

indirect hints that can potentially indicate involvement of DRP2s and SH3P2 in this process as well. Although, there are a number of publications targeting the existence of CIE in plants, they often use indirect approaches and many more in-depth studies need to be performed to unravel the precise mechanism of this process and they did not suggest role of SH3Ps in CIE. Nonetheless, we added some references for the root hair tips and we changed our statement to following:

"… it may be speculated that SH3P2 plays a role in the CIE mechanism, which remains much less characterized in plants (De Angelis et al., 2023; Li et al., 2012; Yu et al., 2020); nonetheless, more research needs to be dedicated to unravel the exact mechanism of such potential pathway and cargoes, that can be internalized through it."

Second decision letter

MS ID#: JOCES/2023/261720

MS TITLE: Role of Dynamin-Related Proteins 2 and SH3P2 in Clathrin-Mediated Endocytosis in *Arabidopsis thaliana*

AUTHORS: Nataliia Gnyliukh, Alexander Johnson, Marie-Kristin Nagel, Aline Monzer, David Babic, Annamaria Hlavata, Saqer S. Alotaibi, Erika Isono, Martin Loose, and Jiri Friml

ARTICLE TYPE: Research Article

I am happy to tell you that your manuscript has been accepted for publication in Journal of Cell Science, pending standard ethics checks. I am sorry that it has taken so long to communicate this to you. Unfortunately we have had some difficulty contacting one of the previous reviewers. That said, the other previous reviewers is now happy with your revisions and I have looked over everything myself and am satisfied that you have dealt with suitably the comments raised. Thank you for your detailed response.

Reviewer 1

Advance summary and potential significance to field

This wrok presents some compelling evidence that in plants DRP2 recruitment to the site of endocytosis is independent of the SH3P2.

Comments for the author

All my concerns have been well adressed. I am happy with the current revision.

© 2024. Published by The Company of Biologists under the terms of the Creative Commons Attribution License (https://creativecommons.org/licenses/by/4.0/).
